# Ecophysiological behavior of major *Fusarium* species in response to combinations of temperature and water activity constraints

**DOI:** 10.1128/aem.01832-24

**Published:** 2025-06-10

**Authors:** Marie-Anne Garcia, Rémi Mahmoud, Marie-Odile Bancal, Pierre Bancal, Stéphane Bernillon, Laetitia Pinson-Gadais, Florence Richard-Forget, Marie Foulongne-Oriol

**Affiliations:** 1 INRAE, MYCSA, Villenave d'Ornon, France; 2 Université Paris-Saclay, INRAE, AgroParisTech 124109, EcoSys, Palaiseau, France; 3 Institut Agro, Univ Rennes, CNRS, IRMAR-UMR 6625, Rennes, France; Anses, Maisons-Alfort Laboratory for Food Safety, Maisons-Alfort, France

**Keywords:** fungal pathogens, climate change, ecological niche, adaptive response, inter/intraspecific diversity, combined effects

## Abstract

*Fusarium* head blight (FHB) is a devastating fungal disease affecting cereals, caused by *Fusarium* species that can produce harmful mycotoxins. *Fusarium* species coexist within the same ecological niche during infection, with their population dynamics and associated mycotoxin patterns strongly influenced by the environment. This study provides a comprehensive investigation of the ecophysiological responses of the major *Fusarium* species causing FHB under varying abiotic factors. We assessed growth and mycotoxin production of different isolates of *Fusarium avenaceum, Fusarium graminearum*, *Fusarium langsethiae, Fusarium poae,* and *Fusarium tricinctum* under 24 combinations of temperature (θ = 15, 20, 25, 30°C) and water activity levels (*a*_*w*_ = 0.99, 0.98, 0.97, 0.96, 0.95, 0.94). Our findings indicated that θ, *a*_*w*_, and their interaction have a main significant impact on species behavior. Thanks to innovative statistical approaches using fungal growth data from optical density measurements and mycotoxin quantification, we demonstrated significant inter- and intra-specific differences in environmental responses. Growth and mycotoxin production of *F. graminearum* and *F. avenaceum* appeared favored under high temperature (≥25°C) and high water activity (≥0.97), whereas lower *a*_*w*_ levels (≥0.95) were also conducive for *F. poae* and *F. tricinctum*. A specific and unique behavior of *F. langsethiae* to lowest temperatures (≤20°C) was highlighted. Understanding the ecophysiological requirements of *Fusarium* species is crucial in the context of climate change, which is expected to worsen disease outbreaks. This study provides valuable knowledge for improving the reliability and robustness of FHB prediction models and anticipating the associated mycotoxin risk.

**IMPORTANCE:**

*Fusarium* species pose a significant threat to major cereal crops, particularly wheat, by reducing yields and producing mycotoxins that are harmful to animals and humans. The prevalence of each *Fusarium* species is strongly influenced by environmental conditions, and climate changes have already been reported as responsible for shifts in pathogen populations, leading to changes in mycotoxin patterns. This study revealed distinct ecophysiological behaviors, including growth and mycotoxin production, of the five major *Fusarium* species infecting small grain cereals when exposed to varying temperature and water activity conditions. Our findings provide a valuable foundation for a deeper understanding of mycotoxin risk and for developing more effective mitigation strategies in the near future.

## INTRODUCTION

*Fusarium* head blight (FHB) is a devastating fungal disease affecting small grain cereals worldwide. FHB is mainly caused by several *Fusarium* species that can produce various mycotoxins harmful to humans and animals. In addition to economic losses due to reduced yield and lower grain quality, mycotoxin-producing *Fusarium* species also induce important waste due to the downgrading of contaminated batches. Among the species causing FHB in Europe, *Fusarium graminearum* is acknowledged as the key actor. But *F. graminearum* is never found alone and commonly co-occurs with other *Fusarium* species during infection. *Fusarium avenaceum* and *Fusarium tricinctum* are frequently found associated with FHB (1–6). Recent works also highlighted the increased occurrence of other species in Europe such as *Fusarium poae* or *Fusarium langsethiae* (4–7). Each *Fusarium* species has its own mycotoxin profile. *F. graminearum* is a well-known producer of type B trichothecenes (TCTB) including deoxynivalenol (DON) and its acetylated derivatives (3-acetyldeoxynivalenol [3-ADON] and 15-ADON), nivalenol (NIV) and zearalenone (ZEA) (8). *F. langsethiae* and *F. poae* produce type A trichothecenes (TCTA) including diacetoxyscirpenol (DAS), T-2, and HT-2 (9, 10), but *F. poae* can also produce TCTB (NIV and fusarenon-X [FX]) as well as enniatins (ENN). *F. avenaceum* and *F. tricinctum* produce enniatins (enniatin A [ENNA], ENNA1, ENNB and ENNB1) and beauvericin (BEA) (11). All these *Fusarium* mycotoxins are known for their cytotoxic effect (12), especially TCTB and TCTA, which are reported to be immunosuppressive and inhibit the synthesis of proteins in eukaryotes (13–18). ZEA is estrogenic, i.e., it affects fertility and reproduction (19). The toxic effects of ENN and BEA are related to their ionophoric properties and therefore their capacity to affect the integrity of biological membranes (20). In Europe, most of these mycotoxins are covered by regulations fixed by the European legislation. Maximum levels and guidance values for mycotoxins in cereals are constantly evolving due to new knowledge in toxicology and occurrence data, as well as shifts in FHB pathogen populations associated with changes in mycotoxins contaminating crops (21).

Climate change is likely to deeply impact global food safety. One of the major concerns is the increasing risk of human exposure to mycotoxins (22, 23). Several studies have already demonstrated that climate change has led to substantial fluctuations in the distribution of FHB pathogens and the associated mycotoxin profiles (4, 7). Changes in the prevalence of one *Fusarium* species over the others, together with the subsequent major mycotoxin contaminating harvest, are becoming more common in Europe (4, 8, 24–27). Understanding the biological and climatic factors influencing *Fusarium* populations is crucial for effectively managing FHB and mycotoxin risks.

While *Fusarium* species are likely to share the ecological niche and face similar biotic and abiotic pressures during plant infection, prior research suggests subtle differences in their inherent ecophysiological needs. These variations may translate into differential adaptive capacities under fluctuating environmental conditions, ultimately favoring the establishment of the most fit species. Furthermore, the extensive array of secondary metabolites, including mycotoxins, produced by *Fusarium* species can provide a significant competitive edge during niche colonization (28, 29). Beyond the restricted phase of host plant interaction, the interplay of ecophysiological requirements and environmental constraints may drive a temporal and dynamic shift in *Fusarium* species’ niche occupation across their life cycle.

Several studies have investigated the *in vitro* responses of the predominant *Fusarium* species under abiotic constraints, mainly temperature (θ) and water activity (*a*_*w*_), with a particular focus on growth and mycotoxin production (30–32). Overall, those studies suggested that each species exhibits a distinctive response signature and that within a given species, optimal conditions required for mycelium growth and mycotoxin production are most often different. For example, 25°C is the optimal temperature for growth and DON production by *F. graminearum* (30), while *F. langsethiae* produces HT-2 and T-2 optimally between 15 and 35°C (31). Knowledge about the way that abiotic parameters modulate fungal development and mycotoxin production of minor *Fusarium* species is much more limited (11). Besides, the currently available data have been obtained using quite different experimental conditions (culture media or abiotic factors), mostly using only one or two isolates per species, which does not allow us to assess the adaptation capacity of the different species and speculate which species will be more suited to a particular environment.

To address the aforementioned knowledge gaps, the present work aimed to study the combined effect of temperature and water activity on growth and mycotoxin production, considering the five most prevalent *Fusarium* species causing FHB, with a focus on inter- and intra-specific variability. Using 24 *in vitro* combinations of temperature and water activity (four temperatures—15, 20, 25 and 30°C—combined with six *a*_*w*_ levels: 0.99, 0.98, 0.97, 0.96, 0.95, and 0.94), we finely characterized the growth and mycotoxin production capacity of five isolates for each of the five studied *Fusarium* species (*F. avenaceum*, *F. graminearum*, *F. langsethiae*, *F. poae,* and *F. tricinctum*). Data were statistically analyzed to the contribution of abiotic factors but also of inter- and intra-specific diversity to species behavioral outcomes.

## RESULTS

### Influence of environmental conditions on growth

#### Frequency of growth and growth parameters in response to *a*_*W*_ and θ factors

**Fig 1:**
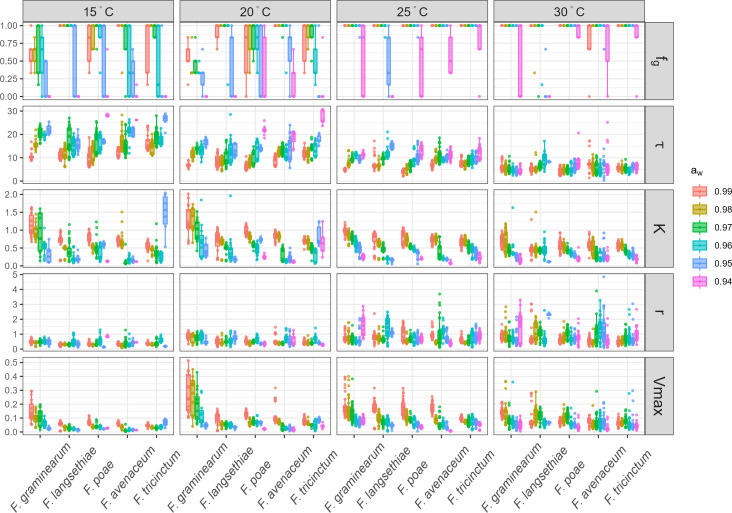
Growth frequency (*f*_*g*_) and growth parameters (𝜏, K, *r*, and Vmax) for the five *Fusarium* species, according to environmental variation. *f*_*g*_ (expressed without unit) and growth parameters (𝜏 expressed in days, K without unit, *r,* and Vmax in OD_unit_.days^−1^) in lines were estimated on five different isolates per species (*F. avenaceum*, *F. graminearum*, *F. langsethiae*, *F. poae,* and *F. tricinctum*, on the *x*-axis). Four different θ (15, 20, 25, and 30°C, shown in columns) and six levels of *a*_*w*_ (0.94, 0.95, 0.96, 0.97, 0.98, and 0.99) were tested. Each dot represents the mean frequency of the six replicates for each isolate, and the boxplots show the intra-specific diversity within the five species.

Growth frequency (*f*_*g*_) and growth parameters (the time at the inflection point, 𝜏, the carrying capacity, K, the relative growth rate, *r,* and the maximal growth rate, Vmax) estimated under different *a*_*w*_ and θ variations for the five isolates of the five studied *Fusarium* species are shown in Fig. 1. Overall, data evidenced that, when growth occurred, the K, 𝜏, *r*, and Vmax parameters followed different patterns depending on the species, isolates, and the environmental factors (*a*_*w*_, θ).

When considering first the *f*_*g*_, highest values (reached *f*_*g*_ = 1) were obtained at θ ≥ 25°C and *a*_*w*_ > 0.94, whatever the *Fusarium* species. Overall, fungal growth was occurring in 2,672 out of the 3,600 inoculated wells. Considering all species and conditions, *F. poae* and *F. avenaceum* exhibited the highest number of wells with detected fungal growth (80%), while *F. graminearum* was the least successful (66%). The highest heterogeneity in *f*_*g*_ was observed for the six *a*_*w*_ levels, at θ ≤ 20°C (Fig. 1). *f*_*g*_ data reported in Fig. 1 also revealed the intra-specific variation of *Fusarium* in responses to environmental changes.

Concerning the 𝜏_mean_ parameter, values determined for all studied *Fusarium* species and isolates were, on average, the highest at 15°C (𝜏_mean_ = 15.8 days). These values gradually decreased when temperature increased to reach an average of 5.76 days at 30°C. In Fig. 1, it could also be noticed that, with few exceptions, decreasing *a*_*w*_ led to an increase in 𝜏_mean_. This pattern was particularly apparent for the 15°C, 20°C, and 25°C conditions.

For most of the studied *Fusarium* species, K values were highest at 20°C or 25°C (Fig. 1). The highest K values were observed for *F. graminearum*. Overall, K was shown to decrease with *a*_*w*_. For example, at θ = 25°C, a fourfold decrease in the K value of *F. graminearum* was observed when *a*_*w*_ decreased from 0.99 to 0.94 (K = 0.98, 0.83, 0.78, 0.62, 0.45, and 0.24 at *a*_*w*_ = 0.99, 0.98, 0.97, 0.96, 0.95, and 0.94, respectively; Fig. 1). Surprisingly, very high K values were fitted for *F. tricinctum* at quite challenging environmental conditions, i.e., K = 1.46 at θ = 15°C, *a*_*w*_ = 0.95 and K = 0.66 and 0.84 at θ = 20°C, *a*_*w*_ = 0.94 and 0.95, respectively. However, such an observation has to be considered as a mathematical artifact since under the former conditions, *F. tricinctum* showed a delayed growth (𝜏 > 25 days) exceeding the duration of the experiment. Indeed, the fitted K could not be seen as indicative of observed growth when the 𝜏 value was too high (𝜏 > 25 days), as maximal optical density (OD) was never reached during the experimentation (Fig. 1 and 3).

Regarding the *r* parameter, its value significantly increased (*P*-value <0.05) with θ (*r*_mean_ = 0.37, 0.53, 0.77, and 0.82 OD_unit_.days^−1^ (ODU.D^−1^) at 15, 20, 25, and 30°C, respectively; Fig. 1). Moreover, for growth conditions with *a*_*w*_ <0.97, no significant difference between *r* parameter values determined for each *Fusarium* species was observed (*P*-value >0.05).

Whatever the considered species, the Vmax parameter globally increased with θ, until 25°C, while a slight decrease was noted at 30°C (Vmax_mean_ = 90 and 75 mODU.D^−1^ at 25°C and 30°C, respectively). Among all the studied species, *F. graminearum* was the fastest-growing species with Vmax_mean_ = 97, 198, 122, and 92 mODU.D^−1^ at 15, 20, 25, and 30°C, respectively (Fig. 1). On the contrary, *F. avenaceum* and *F. tricinctum* were the slowest-growing species with Vmax_mean_ = 74 and 72 mODU.D^−1^ at 25°C or Vmax_mean_ = 32 and 37 mODU.D^−1^ at 15°C, respectively. Overall, the Vmax parameter increased slightly with *a*_*w*_. Interestingly, at θ = 20°C and 25°C, the Vmax values assessed for *F. graminearum* showed a fourfold and twofold decrease, respectively, between *a*_*w*_ = 0.99 and 0.94.

#### Probability of growth: species exhibit contrasted responses to *a*_*W*_ and θ factors

**Fig 2:**
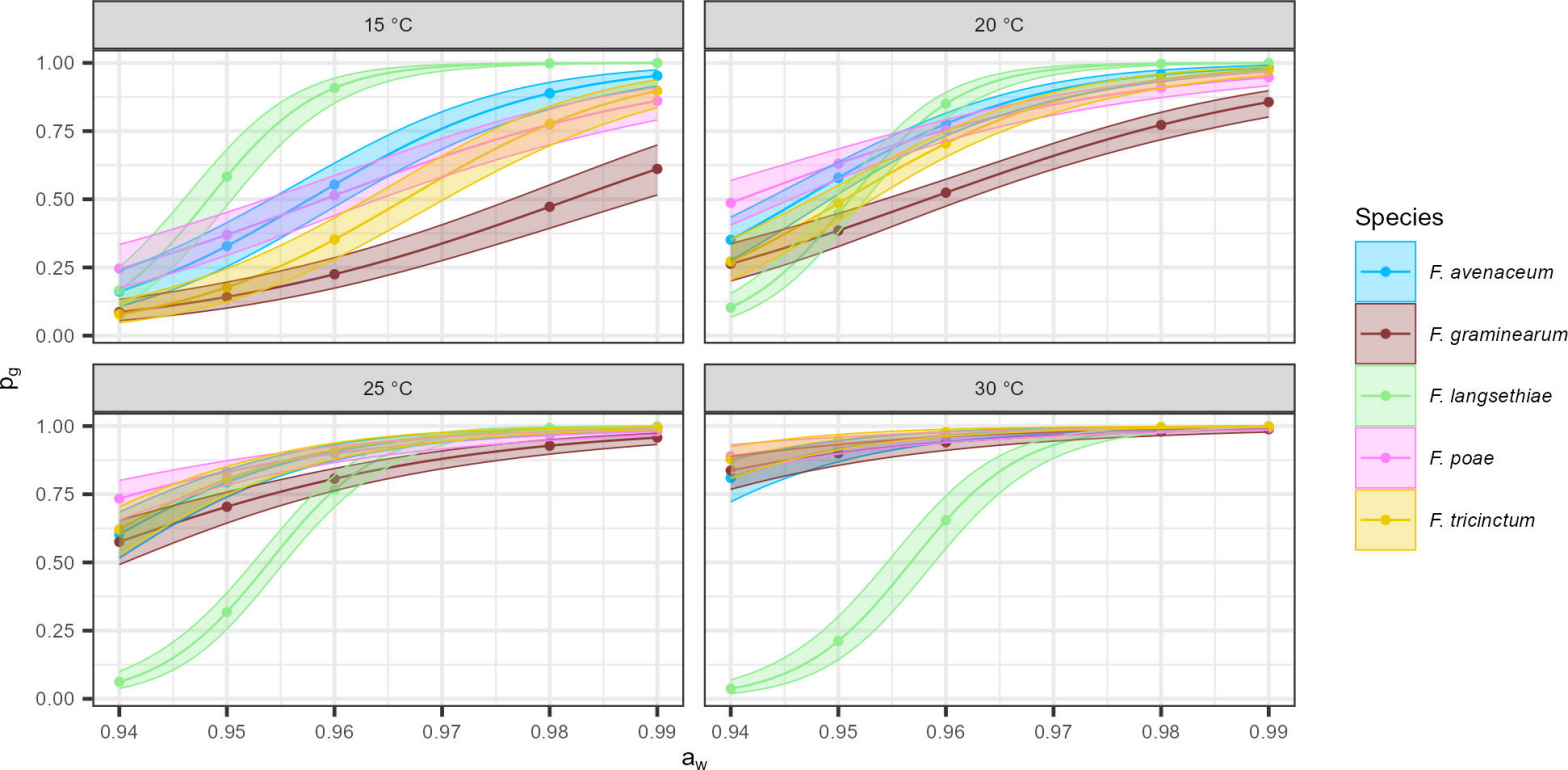
Modeling growth probability with logistic regression for the five *Fusarium* species, according to environmental variation. Growth probability (*p*_*g*_ on the *y*-axis) was estimated by the optimal logistic regression model (logit(*p*_*g*_) = β_0_ + (β_1_ + β_1i_) · θ_i_ + (β_2_ + β_2i_) · *a*_wi_ + β_3i_ · fs_i_) against four θ (15, 20, 25, and 30°C, on each box) and at six levels of *a*_*w*_ (0.94, 0.95, 0.96, 0.97, 0.98, and 0.99; on the *x*-axis). Each dot represents the *p*_*g*_ of the five *Fusarium* isolates for each species. Confidence intervals (95%) are represented by shading around the curves.

The five *Fusarium* species exhibited different growth responses to environmental variations. To investigate the specific effects of *a*_*w*_ and θ factors on growth parameters, mycelium growth was modeled. The probability model with the lowest Bayesian information criterion (BIC) reached an accuracy of 83%. As shown in Fig. 2, *F. langsethiae* displayed a distinct behavior compared to the other species. Indeed, its growth probability (*p*_*g*_) strongly decreased at low *a*_*w*_ while being minimally affected by θ. *F. langsethiae* appeared more adapted to lower temperature and higher water availability. On the other hand, *p*_*g*_ of the other species varied both with *a*_*w*_ and θ. For *F. graminearum, F. avenaceum, F. poae,* and *F. tricinctum*, *p*_*g*_ exceeded 90% only when θ exceeded 25°C and *a*_*w*_ reached 0.97 (Fig. 2). Moreover, *F. graminearum* appeared as the least robust; at 15°C and 20°C, its growth probability was lower than that of any other *Fusarium* species at all tested *a*_*w*_ levels.

#### Growth parameters of species are differentially driven by the interaction of *a*_*W*_ and θ factors

**Fig 3:**
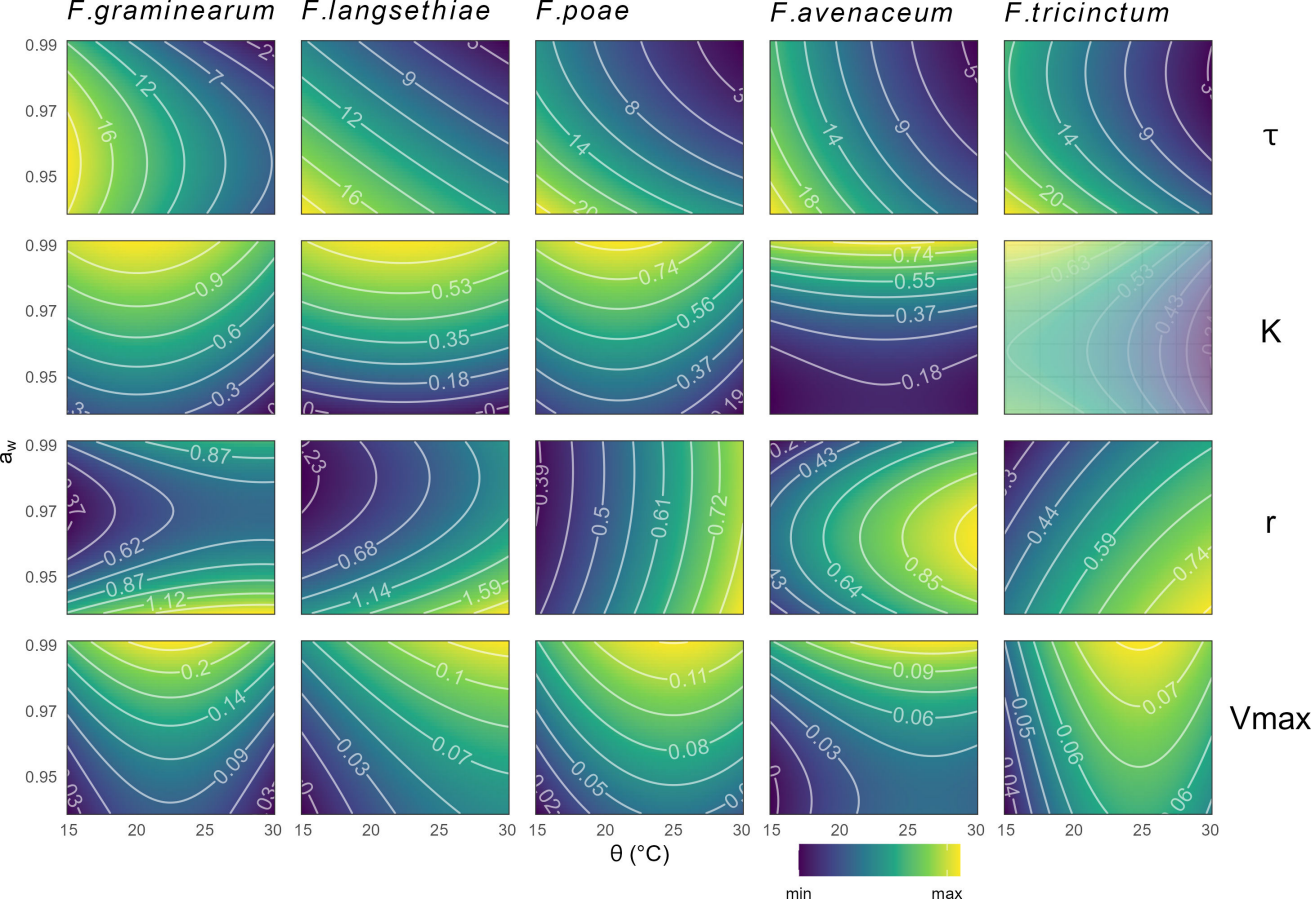
Response surface plots of growth parameters values for the five *Fusarium* species, according to combined abiotic conditions (a_w_ and ϴ). Contour plots of growth parameters (𝜏, K, r and Vmax in line) were estimated on grown replicates using quadratic models for each *Fusarium* species (*F. graminearum, F. avenaceum, F. langsethiae, F. poae* and *F. tricinctum* in column), as a function of a_w_ (y-axis) and ϴ (x-axis). Each response surface has its own scale, this is indicated by numbers on the lines of the contour plot. The contour plot of K for *F. tricinctum* is shaded because the model did not fit correctly the response curves for this species.

Quadratic models, with the parameter as the response variable and *a*_*w*_ and θ as covariates, were applied for each studied *Fusarium* species and allowed us to establish response curves shown in Fig. 3.

Our data indicated a significant increase in time to reach the τ in response to lower temperature (θ ≤ 20°C) and reduced *a*_*w*_, regardless of the species (*P*-value <0.05). Indeed, τ values were maximal at low θ, with little influence of either *a*_*w*_ or species (Fig. 3).

In contrast, the K values were maximal at high *a*_*w*_ levels, with little or no influence of θ (Fig. 3). The quadratic model was unable to correctly fit the K observed values for *F. tricinctum* because of the challenging estimation of this parameter for this species (as described above, in the “Growth parameters and frequency of *Fusarium* species in response to *a*_*w*_ and θ factors” section).

The relative and maximum growth rates also exhibited a different response to *a*_*w*_ and θ depending on the fungal species (Fig. 3). The *r* parameter values assessed for *F. avenaceum*, *F. poae,* and *F. tricinctum* were maximal at θ ≥ 25°C, regardless of the *a*_*w*_. For *F. langsethiae*, the maximum *r* values were observed at θ ≥ 25°C and low *a*_*w*_. The *r* parameter related to *F. poae* showed a dependence on θ only. By contrast, *F. graminearum* showed a complex pattern, with a combined effect of θ and *a*_*w*_ at *a*_*w*_ = 0.97 only, together with a strong effect of extreme *a*_*w*_ (0.95 and 0.99).

The Vmax parameter significantly increased with *a*_*w*_ levels (*P*-value <0.05), with maximal values at high *a*_*w*_, for all the θ or all species (Fig. 3). Temperatures below 20°C were shown to considerably and negatively affect the Vmax parameter for the whole set of studied species.

As shown in Fig. 3, regardless of the considered species, the results showed an increase in 𝜏 and a decrease in K and/or *r* and Vmax rates under challenging conditions. In any case, growth was promoted by *a*_*w*_ ≥ 97%.

#### Inter-specific diversity has only a slight effect on growth parameters, compared to environmental factors

**Fig 4:**
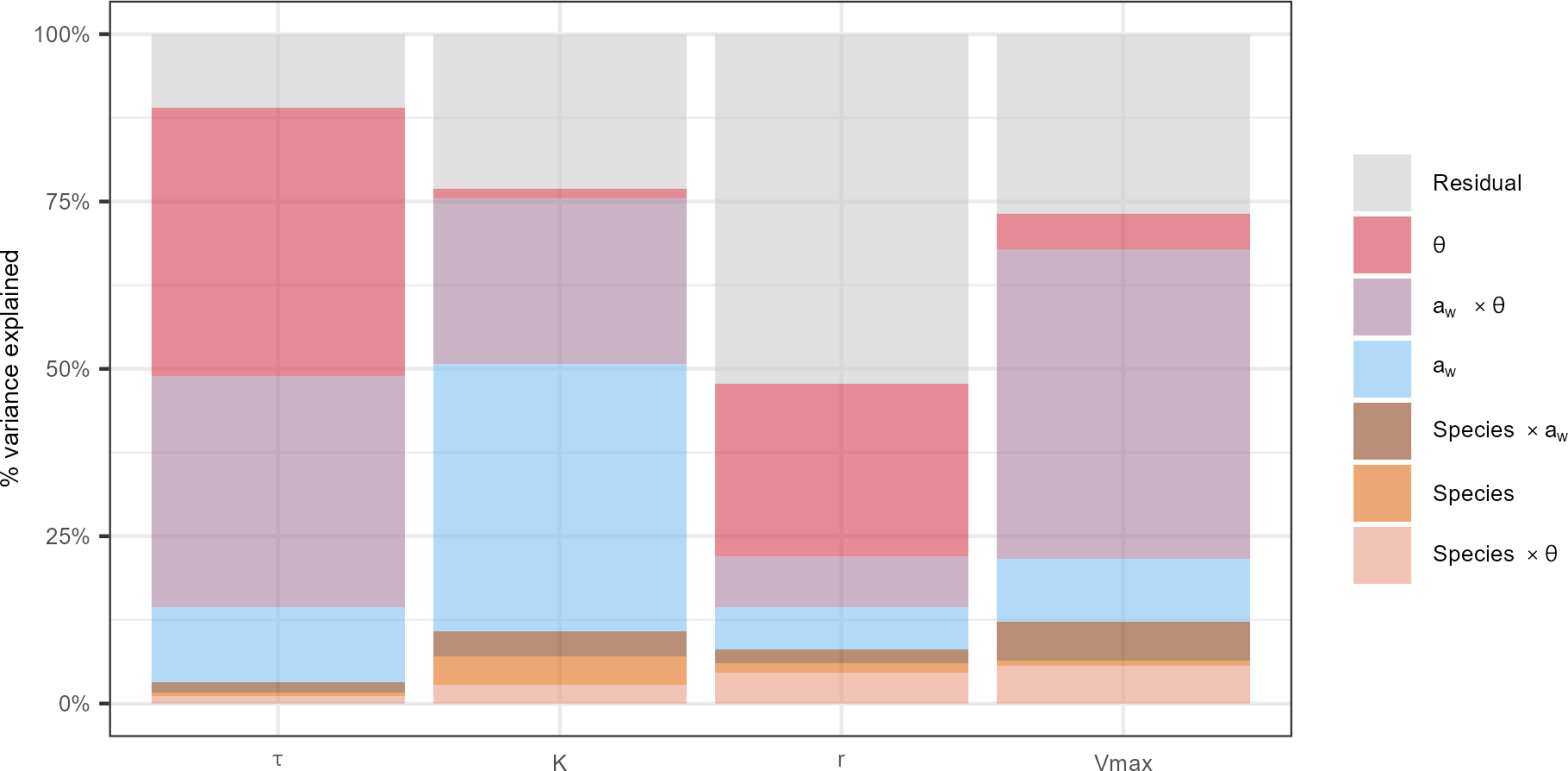
Variance partitioning of growth parameters (𝜏, K, r and Vmax) according to species, environmental factors (a_w_ and ϴ) and their interactions. The percentage of variance explained (y-axis) by different factors (in colors) for each growth parameter (x-axis) was estimated on grown replicates only. Environmental factors (a_w_ and ϴ), their interaction (a_w_ × ϴ), species factor and its interactions with environmental factors (Species × a_w_ and Species × ϴ) are represented in colors. The isolate factor was nested within the species factor.

The above results demonstrated different growth responses between *Fusarium* species. However, how growth variability is distributed between species, isolates, and *a*_*w*_, θ needs further investigation. The variance partitioning represented in Fig. 4 showed that the studied factors (*a*_*w*_, θ, species, and their interactions) explained 89%, 77%, and 73% of the total variance of 𝜏, K, and Vmax parameters, respectively. A different pattern was observed for *r*, with 48% of the total variance explained by the studied factors. The effect of *a*_*w*_, θ and their interaction (a_w_ × θ) on the total variance of each growth parameter was predominant and highly significant (*P*-value <0.05; see Table S1). Nevertheless, the respective contribution of *a*_*w*_, θ and a_w_ × θ to the total variance depends heavily on the measured growth parameter. On the other hand, the species factor alone and its interactions with *a*_*w*_ and θ explained only 3%, 11%, 8%, and 12% of the total variance of 𝜏, K, *r*, and Vmax, respectively; the species factor alone was never significant except for K (see Table S1).

Our data indicated that θ and *a*_*w*_ × θ interaction factors were the main contributors to the variance of the time to reach 𝜏, explaining 40% and 34% of the total variance, respectively, whereas the contribution of *a*_*w*_ factor alone was limited to 11% (Fig. 4; see Table S1).

The variance of the K was explained for 40% and 25% by *a*_*w*_ and *a*_*w*_ × θ interaction, respectively. The θ factor alone contributed only very slightly (less than 2%) to the K variance (Fig. 4; see Table S1).

The main factor contributing to the relative growth rate variance was θ with 26% of the variance explained, followed by *a*_*w*_ and *a*_*w*_ × θ factors that accounted for 6% and 8%, respectively (Fig. 4; see Table S1). But, as mentioned above, half of the variance remained unexplained.

The maximum growth rate variance was shown to considerably depend on *a*_*w*_ × θ interaction that explained 46% of the variance, while *a*_*w*_ and θ factors alone accounted only for 9% and 5%, respectively (Fig. 4; see Table S1).

Altogether, the results reported in Fig. 4 allowed us to identify some trends related to the impact of environmental factors and their interaction on considered growth parameters. The K parameter was mainly affected by *a*_*w*_. 𝜏 and *r* were mainly dependent on θ, while the *a*_*w*_ × θ interaction contributed importantly to the variance of all parameters.

Surprisingly, the species factor and its interactions with abiotic factors contributed only to a low percentage of the variance regardless of the growth parameters. However, as the isolate factor was nested within the species factor, the inter-specific and intra-specific effects on variance were not distinguishable.

#### Intra-specific diversity affects more growth parameters than inter-specific diversity

**Fig 5:**
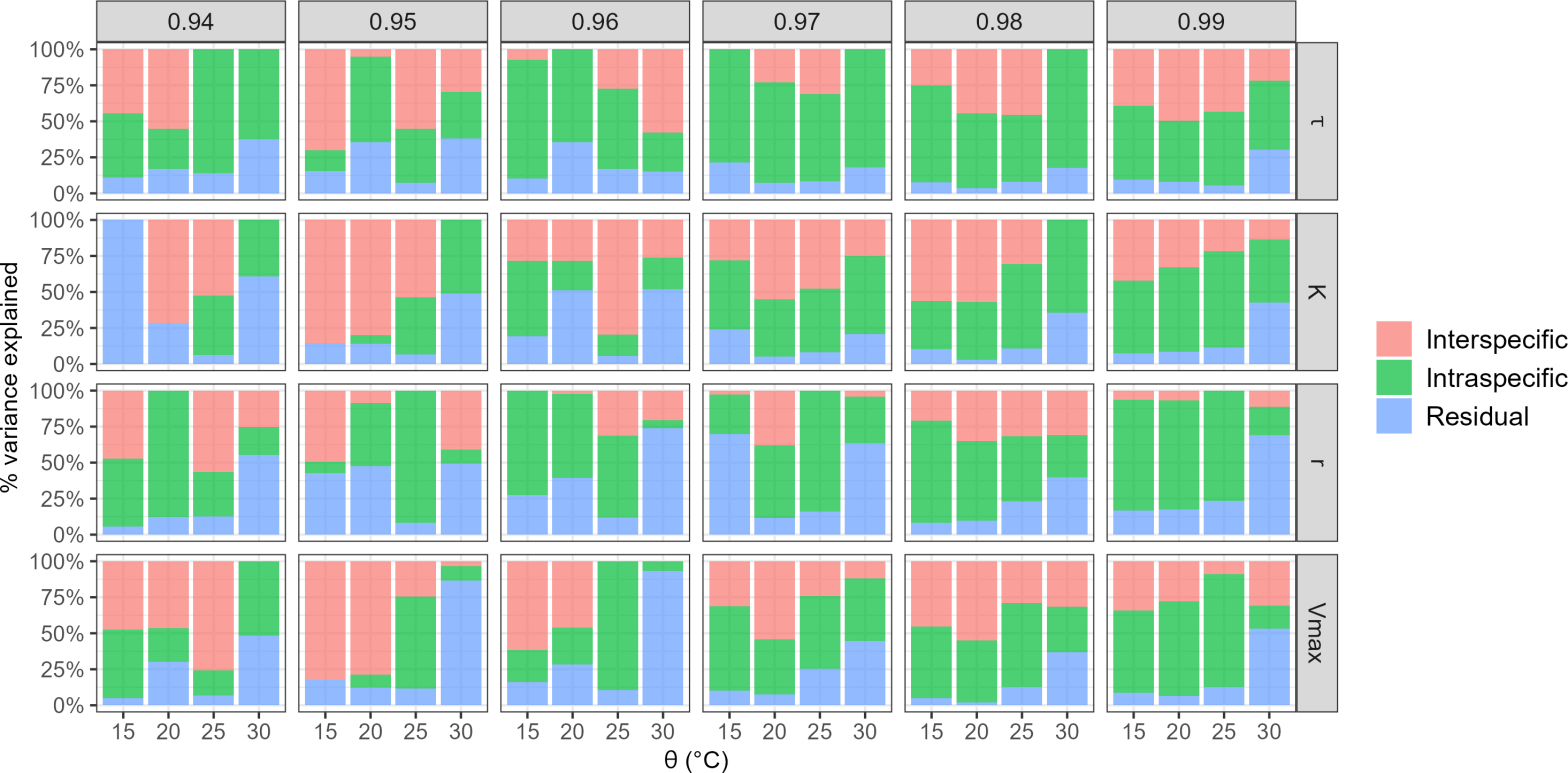
Variance partitioning of growth parameters (𝜏, K, r and Vmax) according to intraspecific and interspecific diversity. Four different ϴ (15, 20, 25 and 30°C, x-axis) and six levels of a_w_ (0.94, 0.95, 0.96, 0.97, 0.98 and 0.99, in column) were tested to assess the effect of interspecific and intraspecific diversity on the total variance (y-axis).

Figure 5 reports the contribution of both intra-specific (isolate factor) and inter-specific (species factor) variability to the variance of the growth parameters for each of the *a*_*w*_ and θ combinations. For *a*_*w*_ values higher than 0.97, the intra-specific diversity accounted for more than 45% of the total variance, while inter-specific diversity contributed 30%, regardless of the environmental conditions.

In contrast, for *a*_*w*_ values lower than 0.97, intra-specific variability contributed less to the variance of growth parameters, and the effect of the species factor appeared to be more pronounced than the isolate factor. For example, 82% and 79% of the Vmax variance was, respectively, explained by inter-specific variability at *a*_*w*_ = 0.95 and θ = 15°C or θ = 20°C (Fig. 5).

The residual variance, not explained by the species or the isolate, contributed nearly 24% of the total variance, this percentage increasing in the less favorable growth conditions (15°C and 30°C). In some cases, the residual effect was even stronger than intra- or inter-specific effect. For instance, the whole variance of the K parameter was unexplained at θ = 15°C and *a*_*w*_ = 0.94 (Fig. 5). However, as mentioned before, estimating the K parameter becomes challenging under severe abiotic constraints, such as θ = 15°C and *a*_*w*_ = 0.94, because of a high 𝜏 value, preventing the plateau from being reached during the experimentation.

#### Intra-specific effect is different between species

Previous results showed a greater effect of the intra-specific diversity on growth parameters, compared to inter-specific diversity. The distribution of variability in growth parameters was further studied, taking into account both isolate and *a*_*w*_, θ and their interaction (*a*_*w*_ × θ).

**Fig 6:**
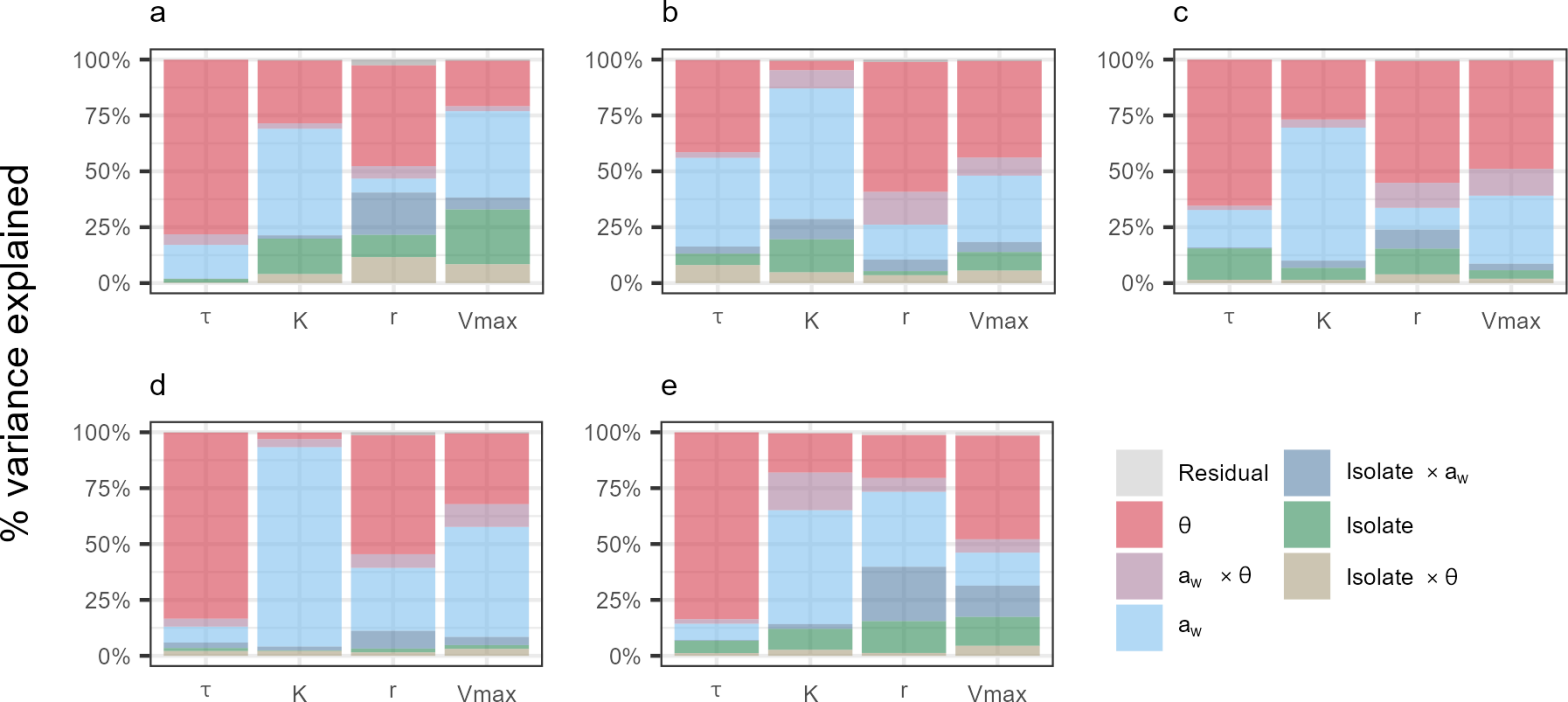
Variance partitioning of growth parameters (𝜏, K, r and Vmax) for the five *Fusarium* species, according to isolate, environmental factors and their interactions. The percentage of variance (y-axis) explained by different factors (in colors) for each *Fusarium* species studied: (a) *F. graminearum*, (b) *F. langsethiae*, (c) *F. poae*, (d) *F. avenaceum* and (e) *F. tricinctum*. Environmental factors (a_w_ and ϴ), their interaction (a_w_ × ϴ), isolate factor and its interactions with environmental factors (Isolate × a_w_ and Isolate × ϴ) are represented in colors.

Intra-specific variance of growth parameters is reported in Fig. 6 for each studied *Fusarium* species. It clearly appeared that this variance was significantly impacted by θ, *a*_*w*_ and the *a*_*w*_ × θ interaction, and that the contribution of the previous factors differed according to the species. Regardless of the species or the studied growth parameter, the proportion of variance explained by the isolate factor and its interactions with abiotic factors never exceeded 40%.

Regarding *F. graminearum*, *a*_*w*_ and θ and their interaction accounted for 98%, 78%, 57%, and 61% of the total variance of 𝜏, K, *r,* and Vmax parameters, respectively, while the isolate factor and its interactions contributed to 2%, 21%, 40%, and 38% (Fig. 6a; see Table S2). This predominant effect of *a*_*w*_, θ and *a*_*w*_ × θ was particularly evident for the τ parameter. By contrast, the isolate factor and its interactions with *a*_*w*_ and θ contributed to a higher percentage of the variance for *r* and Vmax parameters.

When growth parameters of *F. langsethiae* followed globally the same pattern as those related to *F. graminearum*, i.e., *a*_*w*_, θ and *a*_*w*_ × θ strongly contributing to the variance, regardless of the growth parameter (83%, 71%, 88%, and 81% for τ, K, *r* and Vmax, respectively; Fig. 6b; Table S3), the isolate factor and its interactions accounted for a large part of the variance. This was particularly the case for K and Vmax, for which 29% and 18% of their variance was explained by the isolate factor and its interactions, respectively (Fig. 6b; see Table S3).

Considering *F. poae,* environmental factors and their interaction contributed to 84%, 90%, 75%, and 91% of the variance of τ, K, *r*, and Vmax parameters, respectively, underlining a small contribution of the isolate factor and its interactions (Fig. 6c; see Table S4). Indeed, these last factors never explained more than 24% of the total variance, regardless of the parameter.

In the case of *F. avenaceum,* growth parameter variance strongly depended on environmental factors and their interaction. Indeed, these factors accounted for 94%, 96%, 87%, and 91% of the variance of 𝜏, K, *r*, and Vmax, respectively (Fig. 6d; see Table S5). Moreover, the isolate factor and interactions contributed only to 6%, 4%, 11%, and 8% of the variance (Fig. 6d; see Table S5).

For *F. tricinctum*, *a*_*w*_, θ and *a*_*w*_ × θ contributed to 93%, 85%, 59%, and 67% of the variance of τ, K, *r,* and Vmax, respectively, whereas the isolate factor and its interactions with *a*_*w*_ and θ accounted for 7%, 14%, 40%, and 31% (Fig. 6e; see Table S6). The predominant effect of environmental factors and their interaction was particularly observed for τ, while *r* and Vmax parameters were also greatly influenced by the isolate factor and its interactions.

Overall, environmental factors and their interaction on growth parameters were characterized by a higher impact than the isolate factor regardless of the studied species. However, the weight of intra-specific variation was different between *Fusarium* species. Indeed, the intra-specific effect was particularly pronounced on growth parameters of *F. graminearum* and *F. tricinctum,* while *F. langsethiae* and *F. poae* were less impacted. On the other hand, the intra-specific effect had almost no effect on growth parameters of *F. avenaceum*.

### Influence of environmental conditions on mycotoxin production

#### The capacity of species to produce mycotoxin is differentially impacted by *a*_*W*_ and θ factors

**Fig 7:**
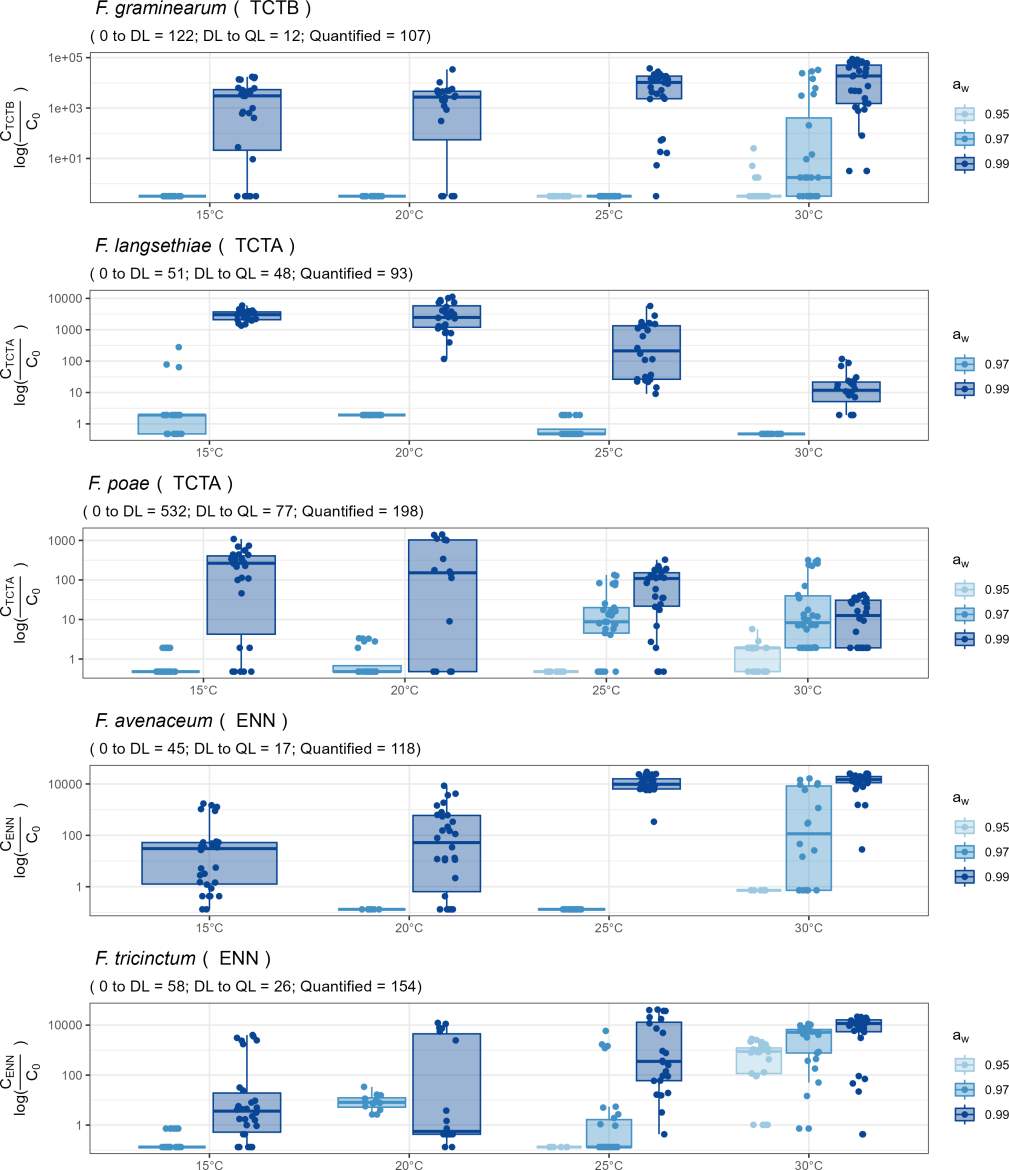
Mycotoxin production by the five *Fusarium* species under environmental variation. TCTB for *F. graminearum* are represented in box 1, TCTA for *F. langsethiae* and *F. poae* in boxes 2 and 3, respectively, and ENNs for *F. avenaceum* and *F. tricinctum* in boxes 4 and 5, respectively. Mycotoxins produced (log-transformed, y-axis) were analysed at four ϴ (15, 20, 25 and 30°C, x-axis) and three a_w_ levels (0.95, 0.97 and 0.99, in colors). Some replicates did not produce mycotoxins at a_w_ = 0.95. Each dot represents one replicate and the boxplots show the intraspecific diversity in terms of mycotoxin production within each *Fusarium* species. 0 to DL corresponds to the number of grown replicates in which the mycotoxins were not detected (concentration below the limit of detection (DL)). DL to QL corresponds to the number of grown replicates in which the mycotoxins were detected but not quantified because they were below the lower limit of quantification (QL). Quantified corresponds to the number of grown replicates where the mycotoxins were quantified.

Figure 7 showed that our experimental conditions allowed the production of measurable amounts of mycotoxins by each of the studied species and isolates for *a*_*w*_ values higher than 0.95, with the exception of some replicates of *F. avenaceum*, *F. graminearum*, *F. poae,* and *F. tricinctum*, grown at 25°C or 30°C.

All isolates of *F. graminearum* were able to produce TCTB but with a large intra-specific variability (see Fig. S3). At *a*_*w*_ = 0.99, data reported in Fig. 7 clearly indicated that *F. graminearum* produced more TCTB as the θ raised. Only the 30°C temperature allowed the production of TCTB at *a*_*w*_ = 0.97 and 0.95, the amount of mycotoxins strongly decreasing at low *a*_*w*_ (Fig. 7).

All *F. langsethiae* isolates were shown to produce TCTA, with the exception of the I508 isolate. This isolate was also characterized by weak growth compared to the other isolates (data not shown). T-2 was the major produced mycotoxin (78% of the total TCTA), while DAS (14% of TCTA) and HT-2 (8% of TCTA) were quantified in smaller amounts. T-2, HT-2, and DAS were detected at *a*_*w*_ = 0.99 under all the tested temperature conditions, the highest amounts being observed at low θ (15 and 20°C, Fig. 7). At *a*_*w*_ = 0.97, a low level of TCTA was quantified at 15°C only.

Regarding *F. poae*, isolates of this species, cultivated in our experimental conditions, were able to produce mycotoxins belonging to three families (see Fig. S3): TCTB (FX), TCTA (DAS and T-2), and ENN (ENNB and ENNB1). A significant correlation (⍴ = 0.8, *P*-value <0.05) was observed between TCTB and TCTA, but none with ENN (for example, between TCTA and ENN, ⍴ = 0.33, *P*-value = 0.38). Indeed, the conditions promoting the production of ENN differed from that of TCTs. DAS was the sole mycotoxin produced above the limit of quantification (QL) by the five *F. poae* isolates at all θ. When the *a*_*w*_ was set at 0.99, the highest production of DAS was observed at low θ (15°C and 20°C, Fig. 7), while at *a*_*w*_ = 0.97 and 0.95, the amount of DAS increased with θ (*a*_*w*_ = 0.97 at 20, 25, and 30°C; and *a*_*w*_ = 0.95 at 30°C).

All isolates of *F. avenaceum* and *F. tricinctum* produced ENNs in the experimental conditions used throughout this study, the produced amounts varying according to the isolate. ENNB (representing 90% and 60% of total ENNs for *F. avenaceum* and *F. tricinctum*, respectively) and ENNB1 (9% and 32% of total ENNs) were the predominant quantified mycotoxins, whatever the θ or *a*_*w*_ (see Fig. S3). These two species tended to produce more ENNs as the θ raised (Fig. 7). The highest levels of ENN were quantified at *a*_*w*_ = 0.99, 25°C and 30°C. Some ENNs were also quantified in cultures of *F. avenaceum* at *a*_*w*_ = 0.97 but only at 30°C (Fig. 7). In contrast, the conditions allowing the production of ENNs by *F. tricinctum* were less stringent since measurable amounts of ENNs were observed at *a*_*w*_ = 0.97 under 20, 25, and 30°C, and even at *a*_*w*_ = 0.95 and 30°C.

#### Probability of mycotoxin production according to environmental factors and species

**Fig 8:**
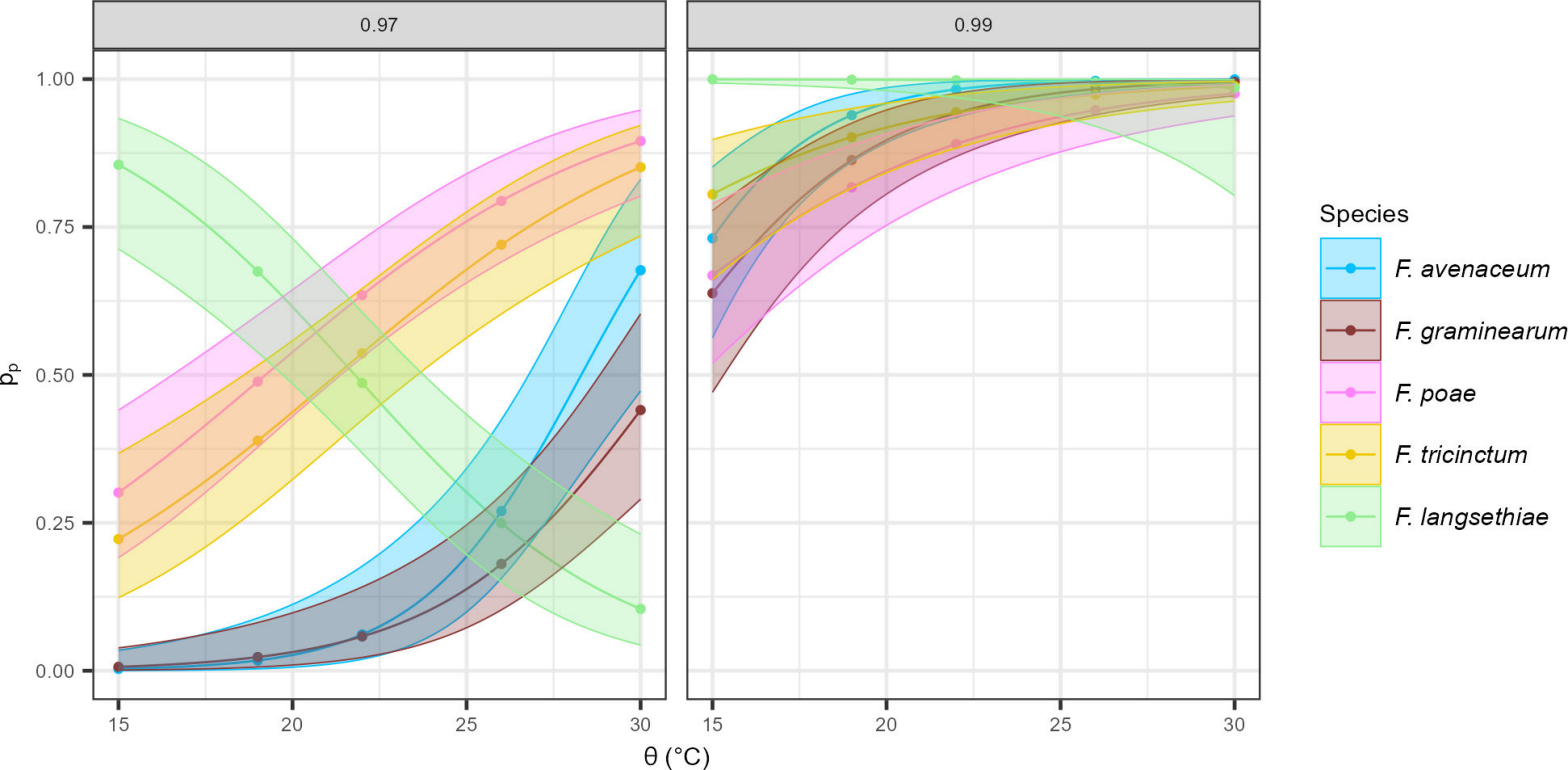
Probability of mycotoxin production for the five *Fusarium* species, according to environmental variation. The probability of mycotoxin production (y-axis) was estimated by the logistic regression model (logit(p_p_) = β_0_ + (β_1_ + β_1i_)·ϴ_i_ + β_2i_·a_wi_ + β_12i·_a_wi_·ϴ_i_ + β_3j_·fs_j_ + β_ij_·a_wi_·fs_j_) against four ϴ (15, 20, 25 and 30°C, on the x-axis) and at two levels of a_w_ (0.97 and 0.99, in column). Each dot represents the probability of mycotoxin production of the five *Fusarium* isolates for each species. Confidence intervals (95%) are represented by shading around the curves. Data for a_w_ = 0.95 are not shown because very few grown replicates were able to produce mycotoxins above the quantification threshold.

Previous results showed that the five *Fusarium* species studied in the present work have different abilities for mycotoxin production. The frequency of measurable mycotoxin levels was assessed in the subset of samples incubated at *a*_*w*_ = 0.97 and 0.99 and θ = 15°C, 20°C, 25°C, and 30°C, thus amounting to 699 measurements over 989 grown replicates (see Fig. S4). Mycotoxins were detected in 71% of the total inoculated wells. Only 57% of *F. graminearum* samples contained mycotoxins, compared to 74% for the four other species. Fitting mycotoxin frequency to the logistic regression model indicated that θ, *a*_*w*_ and *Fusarium* species factors had significant effects, as well as their interactions (Fig. 8).

The probability of mycotoxin production (*p*_*p*_) for each studied species is reported in Fig. 8. As a general pattern, *p*_*p*_ increased with *a*_*w*_ and θ. At *a*_*w*_ = 0.99, the probability that a species produces measurable levels of mycotoxins is high, especially for θ > 20°C. However, as previously reported for growth, *F. langsethiae* displayed a distinct behavior compared to the other species. At *a*_*w*_ = 0.97, the probability of mycotoxin production by this species strongly decreased with the θ (Fig. 8). When comparing the five species at *a*_*w*_ = 0.97, it appeared that *F. poae* and *F. tricinctum* were characterized by the highest *p*_*p*_.

A censored two-way analysis of variance (ANOVA) was performed using the whole set of mycotoxin data acquired in the present study (see Fig. S5). This allowed us to clearly determine differences in the effects of θ and *a*_*w*_ on the production of mycotoxins by each of the studied *Fusarium* species. *a*_*w*_ was shown to significantly modulate the mycotoxin production, regardless of the species, while θ had a significant impact only for *F. graminearum, F. langsethiae,* and *F. tricinctum*. This last species was the only one affected for its mycotoxin production by the *a*_*w*_ × θ interaction (see Fig. S5; see Table S7).

#### Correlation between environmental conditions, mycotoxins, and growth parameters

**Fig 9:**
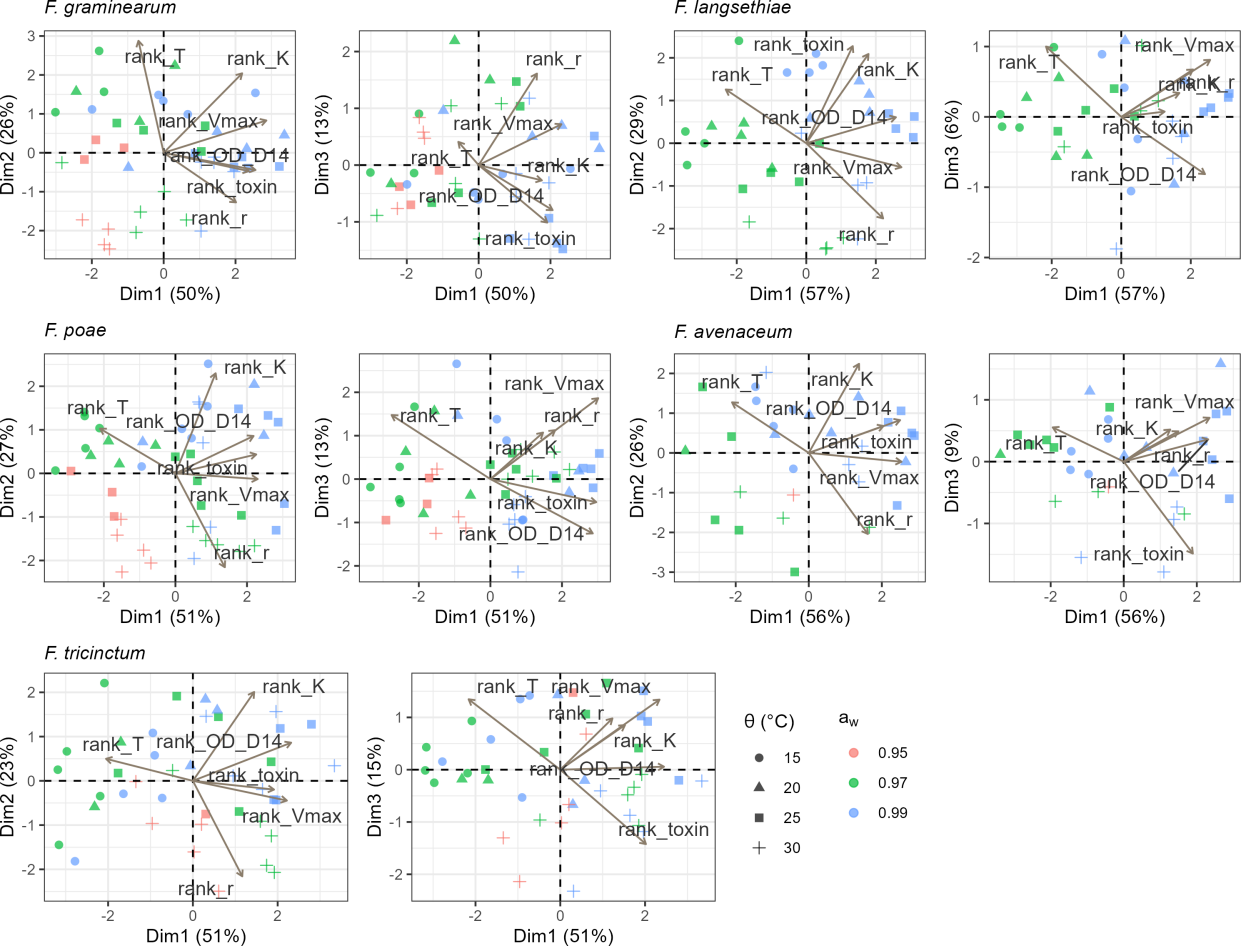
Principal component analysis of mycotoxin and growth parameters (𝜏, K, r and Vmax) for the five *Fusarium* species. The ranks of growth parameter and mycotoxins for *F. avenaceum*, *F. graminearum*, *F. langsethiae*, *F. poae* and *F. tricinctum* are presented at four ϴ (15, 20, 25 and 30°C) and three a_w_ levels (0.95, 0.97 and 0.99). Ranking was performed separately for each group of mycotoxins associated with a species : TCTB (DON + 15-ADON) for *F. graminearum*, TCTA (DAS + T-2 + HT-2) for *F. poae* and *F. langsethiae*, and ENNs (ENNA + ENNA1 + ENNB + ENNB1) for *F. avenaceum* and *F. tricinctum*. For each species, first biplot is for (Dim1, Dim2) and second biplot is for (Dim1, Dim3). Each dot corresponds to an isolate growing at specific a_w_ and ϴ.

Correlations between growth parameters and mycotoxin production were analyzed, considering the studied *a*_*w*_, θ and *a*_*w*_ × θ. All data (ranks of growth parameters and mycotoxin) related to each fungal species were combined in principal component analyses (PCA) (Fig. 9). The first axis represented 50% to 56% of the variance depending on the considered species. For *F. avenaceum, F. graminearum, F. langsethiae,* and *F. tricinctum*, the first axis was shown to discriminate samples characterized by early (τ) and high (Vmax) growth leading to high OD at 14 days, together with high mycotoxin production levels (ρ = 0.39 to 0.57), from late and low growth samples, together with low mycotoxin production levels. The second axis represented 23 to 27% of the variance, leading to a negative correlation between ranks of τ and *r* (ρ = −0.51). The third axis contributed to 6 to 15% of the variance. This axis primarily reflected variations in mycotoxin ranks, which tended to be higher in samples grown at high *a*_*w*_. The mycotoxin ranks moderately correlated with other growth parameters (ρ = 0.31 for K, −0.36 for τ, and 0.13 for *r*) and more strongly with OD at 14 days (ρ = 0.48). The previous observations suggest that samples characterized by delayed growth were associated with lower production of mycotoxins. Overall, results presented in Fig. 9 clearly show that mycotoxin production is correlated to growth parameters, regardless of the species, and differentially driven by *a*_*w*_, θ. Finally, the growth and the mycotoxin production of *F. graminearum, F. avenaceum,* and *F. tricinctum* were correlated and were specifically favored by θ ≥ 25°C.

## DISCUSSION

FHB is a multi-pathogen disease caused by a consortium of several *Fusarium* species frequently occupying the same niche during infection. A more comprehensive understanding of the intricacies of such multiple infections is essential to predict the outcome in terms of disease development and also mycotoxin production. Climate is certainly the first driving force that shapes *Fusarium* spp. occurrence in the field (33). Using *in vitro* studies, we investigated the behavior of major *Fusarium* species under various challenging environmental conditions.

Several studies have already addressed the impact of abiotic constraints on the growth and mycotoxin production of *Fusarium* species (34–36). But to the best of our knowledge, no comprehensive analysis as the one discussed in the present study has been published before. Indeed, this study is the first one considering five major *Fusarium* species causing FHB and a large range of combined temperature and water activity conditions. In addition, whereas most of the earlier studies have mainly focused on one or a couple of isolates, the present one was based on a set of five isolates per species. Lastly, the analytical process of our data set was optimized by the use of innovative statistical approaches.

### Optimizing analyses to fully exploit relevant biological data

Inferring fungal growth rates from OD measurements has already been proven to be relevant, either for yeast-like microorganisms or for filamentous fungi (37, 38). In this paper, the OD readings were directly used as raw data, generating a large data set that allowed robust statistical analyses for modeling growth and estimating time-course growth parameters for each *Fusarium* species. Thus, the observed growth curves were consistent with theoretical growth patterns, displaying the four expected phases: lag, exponential, deceleration, and stationary (see Fig. S1), despite hyphal growth and the possible production of secondary metabolites that could affect OD measurements. The measurements were repeatable across replicates and revealed different responses to environmental conditions. High-throughput phenotyping experiments involving numerous isolates and replicates are known to be time-consuming and highly burdensome while generating substantial waste (39, 40). To reduce such constraints, optimal processing of data is required, necessitating the implementation of appropriate statistical methodologies. Thus, generalized additive models (GAMs), as used in the present work, have been shown to allow an accurate mapping of the OD of the culture medium, improving the sensitivity and specificity of fungal growth and estimated growth curves, even at very low values of OD (41). Furthermore, in the present study, instead of discarding wells lacking growth/mycotoxin production, logistic regression models were applied and have provided valuable insights into the intrinsic dynamics of these wells. As far as we know, this was the first time where growth and mycotoxin production were expressed as probability. Such insights are particularly relevant in the context of FHB, where the presence or absence of species in the field carries significant implications beyond mere quantitative considerations. Moreover, in the analysis of mycotoxins, we decided not to discard values falling below detection/quantitation limits (observed in 46% of the samples) and to not substitute them with arbitrary values, according to practices recommended by Shoari and Dubé (42). By employing methods tailored to handle censored data, we were able to explore a broader spectrum of environmental conditions.

### Differential responses of *Fusarium* species under fluctuating environmental conditions

Growth and mycotoxin production responses to changing environmental conditions were shown to differ according to the studied *Fusarium* species. Our results indicated that *F. graminearum* was the most affected in its growth ability by challenging environmental conditions, suggesting that this species could display a lower phenotyping robustness than the other species. Our data corroborate previous reports indicating the highest growth rates at *a*_*w*_ = 0.99 and θ = 25°C for *F. graminearum* (30).

Consistently, the growth probability assessed for *F. graminearum* was lower than that of the other *Fusarium* species, except at 25°C. This observation could be the result of different ecophysiological requirements of *Fusarium* species, particularly for growth initiation. Indeed, in the literature, 25°C is often considered to be the optimum temperature for *F. graminearum* germination (43).

We showed that *F. langsethiae* exhibited a different behavior compared to *F. graminearum*, *F. avenaceum*, *F. poae,* and *F. tricinctum*. Notably, the highest probabilities of growth and mycotoxin production for this species were observed at 15°C. This distinct behavior is supported by previous ecophysiological studies (32, 44–46). Our findings suggest that *F. langsethiae* may occupy a distinct ecological niche compared to other species or possess a competitive advantage in colonizing niches in colder environments (9), which is consistent with its prevalence in northern regions (47–49). Furthermore, maintaining consistent T-2/HT-2 production at lower temperatures may give *F. langsethiae* a competitive advantage, as these mycotoxins are among the most potent known (16, 50).

We have also observed that combined environmental factors, a_w_ and θ, impacted significantly both the growth dynamics and mycotoxin production, regardless of the species. Our findings showed contrasted responses of 𝜏 and K parameters, suggesting that the growth rate is more affected by θ, while *a*_*w*_ has a greater influence on final growth. Verheecke-Vaessen et al. (32) observed similar results using three *F. langsethiae* isolates, demonstrating that the lag phase was predominantly impacted by θ. Additionally, the growth of *F. graminearum* and *F. culmorum* was reported as being strongly affected by θ variations (30). Our data also demonstrated that mycotoxin production is strongly affected by *a*_*w*_ levels and θ, which is consistent with previous studies (44, 45). The latter authors observed a major effect of *a*_*w*_ on T-2 and HT-2 mycotoxin production by *F. langsethiae*. We specifically observed that the production of mycotoxins by *F. graminearum* was more restrained in challenging conditions than that of the other species, as described by Hope et al. (30).

Moreover, we highlighted the correlation between growth and end-point mycotoxin production, emphasizing the importance of the mycotoxin production dynamics, as described by Verheecke-Vaessen et al. (32). Further analysis investigating the production of mycotoxin by *Fusarium* species for longer incubation times could clarify whether low amounts of mycotoxin result from a delay in production or an inhibition induced by challenging environmental conditions (30).

### Exploring intra-specific diversity of *Fusarium* species

An essential and thus far under-investigated aim of this study was to consider the intra-specific diversity within five major *Fusarium* species causing FHB. In less challenging growth conditions, we found that intra-specific diversity accounted for a high proportion of the variance compared to inter-specific diversity. However, when *a*_*w*_, θ were included in the analysis, it became clear that their combined effects were governing growth variation at the species and isolate levels. This indicates that while intra-specific diversity has a significant impact on *Fusarium* species behaviors, its effect is negligible compared to abiotic factors, particularly in more challenging environments.

Besides, our findings illustrate the extent of the *in vitro* phenotypic responses to abiotic constraints exhibited by each species, suggesting the existence of genotypes that are likely to be better adapted. Our data corroborate previous reports indicating that *Fusarium* species and different isolates within the same species exhibit varying degrees of resilience to environmental fluctuations and that such diversity should absolutely be considered in initiatives aiming to predict the impact of abiotic factors on FHB (30–32). They support the conclusion of Ma et al. (51) that has highlighted the difficulty of predicting FHB risk due to the occurrence of populations of distinct isolates in the field.

### Beyond the ecophysiology of *Fusarium* species: anticipate their future distribution under climate change

Climate change is expected to strongly impact the distribution of *Fusarium* species in Europe (52) and could exacerbate the damages related to FHB, including the contamination of grains with mycotoxins (53). We confirmed that environmental conditions are likely to be a key driver of the predominance of one *Fusarium* species over the others. Despite being derived from *in vitro* experiments and not directly applicable to natural conditions, our results interestingly corroborate field surveys that have documented shifts in *Fusarium* species distribution. For example, Covarelli et al. (25) confirmed the predominant effect of thermo-hygrometric conditions in the replacement of *F. graminearum* by *F. avenaceum* and *F. poae* between 2009 and 2010. Other studies have also shown that the occurrence of *F. graminearum* is promoted by moderately warm summers, whereas dry and hot climates are more favorable to *F. poae* or *F. avenaceum* (4, 8, 26, 27). Predicting the distribution of FHB species and the associated mycotoxin risk is therefore essential to limit disease outbreaks and ensure feed and food safety. Beyond the factors studied here, other abiotic factors that could affect *Fusarium* spp. behavior would warrant investigation. For example, in addition to the impact of elevated atmospheric CO_2_ on *Fusarium* spp., which has already been documented (54–56), the effect of irradiance or circadian rhythm remains poorly understood. Additionally, *Fusarium* species may exhibit different abilities to respond to wheat resistance mechanisms triggered during plant/pathogen interactions. For example, oxidative stress has been identified as a factor influencing mycotoxin biosynthesis pathways of *F. graminearum* (57), highlighting the importance of complementing *in vitro* studies by *in planta* research.

Battilani et al. (58) have established a mechanistic weather-driven model based on the life cycle of *Aspergillus flavus* to predict field contamination by aflatoxins. They emphasized the importance of including ecophysiological parameters of fungal species in the models. A similar conclusion was raised by Van Der Fels-Klerx et al. (59) who have attempted to model climate change effects on wheat phenology and DON contamination in wheat cultivated in North West Europe by 2040. The previous authors have used various climatic and agronomic variables to calculate a principal empirical model underlying the biology of *Fusarium* infection and DON production. The present study highlighted intra-specific and inter-specific ecophysiological properties of *Fusarium* species under environmental variations. These experimental data could be used as variables to calibrate the model parameters and improve the prediction of FHB risk and mycotoxin contamination.

In order to draw a comprehensive pattern of response to abiotic conditions, we investigated the ecophysiology of *Fusarium* species, one by one. Nevertheless, in the field, a cohort of *Fusarium* species co-occurs during FHB infection (60). The composition of this *Fusarium* community changes over time and location and depends both on environmental and agronomic factors (61), influencing the abundance of one species compared to another (62).

Furthermore, in addition to the ecophysiological characteristics of each species, their capacity to compete is also a key criterion governing their occurrence. As an example, *F. graminearum* is described as being one of the most competitive species when interacting with other *Fusarium* species on wheat spikes (29, 63). However, our results suggest that such a dominance could be questioned under challenging conditions. It is now imperative to investigate the ecophysiology of different *Fusarium* species in interaction in order to better understand the influence of abiotic factors on the competition outcome and to construct a more accurate representation of what happens in the field.

### Conclusions

The present study provides new and significant insights into the ecophysiological properties of the main *Fusarium* species causing FHB in response to the combined effects of temperature and water activity. Five species and five isolates per species were studied across 24 different environmental conditions (four temperatures—15, 20, 25, and 30°C—combined with six *a*_*w*_ levels: 0.99, 0.98, 0.97, 0.96, 0.95, and 0.94). The studied *Fusarium* species exhibited distinct ecophysiological behaviors in response to environmental variations, suggesting that they may belong to different ecological niches at various stages of their life cycle. Our data offer a comprehensive foundation for enhancing predictive models of FHB.

## MATERIALS AND METHODS

### Growth and mycotoxin production

#### Fungal isolates collection

Twenty-five isolates, comprising five isolates from five different *Fusarium* species*—F. graminearum, F. langsethiae, F. poae, F. avenaceum,* and *F. tricinctum*—were used in this study (see Table S8). These isolates were single-spore isolated from wheat, maize, or barley grains and have been thoroughly characterized both phenotypically and genetically. They are registered in public biological resource centers or are maintained at the MycSA laboratory.

#### Culture media

All isolates were inoculated in standardized sucrose synthetic liquid medium (MS) containing 20 g.L^−1^ of sucrose, 0.5 g.L^−1^ KH_2_PO_4_, 0.6 g.L^−1^ K_2_HPO_4_, 0.017 g.L^−1^ MgSO_4_, 1 g.L^−1^ (NH_4_)_2_SO_4_, and 0.1 mL.L^−1^ Vogel mineral salts solution. Glycerol (Sigma-Aldrich, Saint-Louis, USA) was added to liquid medium according to the Norrish equation (64) to adjust the *a*_*w*_ values between 0.94 and 0.99. The resulting *a*_*w*_ was controlled using an *a*_*w*_ meter (TESTO 650, Lenzkirch, Germany). A total of 24 conditions of incubation were considered: temperatures (θ) 15, 20, 25, and 30°C combined to *a*_*w*_ = 0.94, 0.95, 0.96, 0.97, 0.98, and 0.99.

#### Spore production

Fungal isolates were grown on potato dextrose agar (Difco, Le-Pont-de-Claix, France) solid medium for 7 days. For each isolate, the spore solution was prepared by inoculating seven agar plugs in 75 mL of carboxymethylcellulose (Sigma-Aldrich, Saint-Louis, USA) liquid medium. The cultures were incubated for 3 days in the dark with shaking (180 rpm) at 25°C before being filtered (40 µm filter) and centrifuged at 4,800 *g* for 20 min. The supernatants were discarded, and the spore pellets were resuspended in 4 mL of MS. The spores were counted on a Malassez cell and diluted to reach a final concentration of 1.33 × 10^6^ spores.mL^−1^. Batches of spores were kept in 50% glycerol (50:50, vol/vol: glycerol:MS medium) at −80°C until inoculation.

#### Inoculation and study of fungal growth

Batches of spores were thawed on the day of inoculation. The experiment was performed once for each isolate that was inoculated in six replicates at a final concentration of 1.66 × 10^4^ spores.mL^−1^ in 200 µL of MS liquid medium in 96-well plates. Half of the wells of each plate were filled with blanks (non-inoculated wells, with MS at the correct *a*_*w*_) to avoid contamination between the wells and to allow data to be corrected from background between experiments if necessary (see “Estimation of the absorption of the plates” section). Plates were sealed with Breath-easy sealing membrane (Diversified Biotech, Boston) to avoid evaporation and incubated in the dark at the selected θ. OD was measured in each well every 24 hours, five time points, at five different spatial positions in each well, using a spectrophotometer Infinite M200 pro (Tecan, Grödig, Austria) set at λ = 630 nm until the growth stationary phase was observed.

#### Study of mycotoxin production

The same inoculation procedure as described in the previous section was used with the following modifications: 24-well plates were used with a final volume of 2 mL of MS, and six wells per plate were used as controls (non-inoculated wells, with MS at the correct *a*_*w*_). Twelve conditions of incubation were performed: 15, 20, 25, and 30°C combined to *a*_*w*_ = 0.95, 0.97, and 0.99. At the end-point time, i.e., after 14 days of incubation, the cultures were centrifuged (20 min at 4,800 *g*, ambient temperature). Supernatants were kept at −20°C until mycotoxin extraction. Mycotoxins were extracted using 3 mL of ethyl acetate as the extraction solvent. Briefly, the samples were shaken for 5 min and centrifuged for 10 min at 4,800 *g* (ambient temperature). Upper phase was recovered and evaporated with a SpeedVac Concentrator SPD210 (Asheville, USA) at 55°C. Then, 200 µL of methanol/water (50:50, vol/vol) was added, and after homogenization with a Vortex mixer, extracts were filtered through a filter syringe (Acrodisc 13 mm mini spike with 0.2 µm wwPTFE; VWR International, Puerto Rico) and kept at −20°C until analysis.

#### Mycotoxins analysis

A Vanquish UHPLC (ThermoScientific, Bremen, Germany) system was used to separate mycotoxins (type B and A trichothecenes and enniatins) on a reversed-phase column Thermo Accucore aQ C18 (2.1 × 100 mm, 2.6 µm; ThermoScientific, Lithuania) maintained at 40°C. Mobile phase consisted of water/methanol (98:2, vol/vol) (eluent A) and methanol/water (98:2, vol/vol) (eluent B), both containing 5 mM of ammonium acetate and 0.1% (vol:vol) acetic acid. Gradient elution was as follows: 2% of B for 0.5 min, 2 to 98% B in 4.5 min, 98% B for 4.7 min, 98 to 2% for 0.1 min, and 2% B for 2.2 min. The flow was kept at 0.4 mL.min^−1^ and the injection volume was 2 µL. Mycotoxins were detected using a Q Exactive Focus mass spectrometer (ThermoScientific, Bremen, Germany). Heated electrospray ionization source was operated in positive mode. Full scan spectra were acquired at a 70 k resolving power in the 150–1,000 *m/z* range. The Orbitrap analyzer was *m/z*-calibrated each week. Nitrogen was used as the sheath and auxiliary gas. The main MS parameters were set as follows: sheath gas flow rate, 50 psi; auxiliary gas flow rate, 13 a.u.; sweep gas flow rate, 0 a.u.; spray voltage, 3.5 kV; capillary temperature, 320°C; S lens RF level, 50%; auxiliary gas heater temperature, 350°C. External calibration was performed at 5, 10, 25, 50, 100, 250, 400, and 500 µg.L^−1^ to quantify TCTB (DON, 3- and 15-ADON, ZEA, NIV, and FX), TCTA (DAS, T-2, and HT-2), enniatins (ENNA, A1, B, and B1), and BEA. Mix standard of TCTB, TCTA, and ZEA in acetonitrile (10 µg.mL^−1^) was purchased from Romer labs (Tulin, Austria), and mix of ENN (A, A1, B, B1) and BEA in methanol (10 µg.mL^−1^) from LIBIOS (Vindry-sur-Turdine, France).

Some mycotoxins were not detected (concentration below limit of detection), others were detected but not quantified (concentration below the lower QL).

The QL was estimated at 5 µg.L^−1^ for ENN quantification and at 25 µg.L^−1^ for TCTB and TCTA.

Mycotoxin production was calculated by the sum of the different compounds within each group: TCTB (DON + 15-ADON), ENN (ENNA + ENNA1 + ENNB + ENNB1) and TCTA (DAS + T-2 + HT-2) and expressed as final concentration in µg.mL^−1^ before log transformation.

### Data analysis

#### Estimation of the absorption of the plates

To avoid potential biases due to the light absorption by plates and to liquid media interference, 48 non-inoculated wells by plate (MS at the correct *a*_*w*_) were used to assess and model in both spatially and temporally terms, the blank absorption. At each time point (every 24 hours), a spatial GAM was applied using the mgcv package (41) to interpolate the OD values across the entire plate. GAM uses splines, which are smooth functions composed of piecewise polynomials, to relate the X and Y positions of each well to the OD values. GAM was chosen for its flexibility, smoothness, and anisotropic nature (ability to vary in different directions; see Fig. S1). After estimation, the GAM-predicted OD values attributed to the microplates (OD_well_) were subtracted from the measured OD values in each inoculated well, yielding:


$$\Delta OD\;=\;OD_{measured}\;-\;OD_{well}.$$

#### Fitting of the growth curves

For each well, ΔOD was fitted for each day (D) using a sigmoidal growth curve, following the equation reported below:


$$\Delta OD=\alpha +\frac{K}{1+e^{-r(\tau -D)}}$$

α is a parameter describing the well-specific noise of the measurements due to medium absorption and has no biological meaning, while K, *r*, and τ are parameters characterizing the kinetics of ΔOD influenced by the combination of θ and *a*_*w*_. K represents the carrying coefficient of the environment, and it is expressed without unit. *r* is the relative growth rate in OD_unit_.days^−1^, and τ is the time of the inflection point of the curve expressed in days (see Fig. S2). The growth rate at inflection time (Vmax expressed in OD_unit_.days^−1^) is obtained as rK/4. We then employed the drm function from the drc package to fine-tune the curves (65).

#### Modeling the probability of growth

Depending on the experimental conditions, some isolates did not grow at all in some replicates, while the other replicates showed measurable growth. Growth probability was first approximated for each species by calculating the *f*_*g*_ (Fig. 1) of the six replicates for each isolate. The *p*_*g*_ (Fig. 2) was then modeled, using a logistic regression.

The logistic regression method assumes a linear relation between the logit of *p*_*g*_ and the covariates; θ, *a*_*w*_, and *Fusarium* species (fs): logit(*p*_*g*_) = log (*p*_*g*_ / (1 − *p*_*g*_)) = β_0_ + (β_1_ + β_1i_) · θ_i_ + (β_2_ + β_2i_) · a_wi_ + β_12_ · a_wi_ · θ_i_ + β_3i_ · fs_i_ where β_1_ and β_2_ correspond to the effect of θ and *a*_*w*_, respectively; β_3i_ corresponds to the effect of *Fusarium* species i; β_1i_ (and β_2i_) corresponds to the interaction between *Fusarium* species and θ (respectively *a*_*w*_); and β_12_ represents the interaction between θ and *a*_*w*_.

The optimal model was selected among all possible combinations of covariates using the BIC. The logistic regression method is valuable as it offers an analytical approach to model the growth probability. The glm function base was used to adjust the logistic regression model.

#### Variance partitioning analysis

To investigate the influence of each factor on each parameter (K, *r*, τ and Vmax), a type III ANOVA, incorporating θ, *a*_*w*_, *Fusarium* species, and their respective interactions as covariates, was applied. The isolate was nested within the *Fusarium* species factor. The results of the ANOVA were used to decompose the variance explained by each of the factors and their interactions. This analysis was done using the anova function, together with the lmer function of the nlme package.

The proportions of variance explained by the species and the isolate for each parameter, at each level of θ and *a*_*w*_, were compared, using the partvar function of the cati package (66). The effect of the intra-specific diversity was investigated by running an ANOVA within each species on each parameter, with θ and *a*_*w*_ as covariates.

#### Modeling the probability of mycotoxin production

Mycotoxin production was not dynamically measured since the procedure used in the present study was destructive (refer to the section “Study of mycotoxin production”).

Depending on experimental conditions, some species/isolates/replicates were shown to produce quantifiable amounts of mycotoxins while others did not. Similarly to what was done for growth probability, logistic regressions were applied to model the probability of mycotoxin production.

The logistic regression method assumes a linear correlation between the logit of the mycotoxin production probability and the covariates; θ, *a*_*w*_, and *Fusarium* species (fs). The optimal model was selected among all possible combinations of covariates using the BIC.

logit(*p*_*p*_) =log (*p*_*p*_/(1 − *p*_*p*_)) = β_0_ + (β_1_ + β_1i_) · θ_i_ + β_2i_ · a_wi_ + β_12i_ · a_wi_ · θ_i_ + β_3j_ · fs_j_ + β_ij_ · a_wi_ · fs_j_ where β_1_ corresponds to the effect of θ, β_1i_ (and β_2i_) corresponds to the interaction between species and θ (respectively *a*_*w*_), β_12i_ represents the interaction between θ and *a*_*w*_, β_3j_ corresponds to the effect of *Fusarium* species j, and β_ij_ corresponds to the interactions between *a*_*w*_ and the *Fusarium* species j.

However, for *F. langsethiae*, numerical issues were faced since the *a*_*w*_ factor induced a separation problem (when a covariate completely explains an outcome [67]; where all samples exhibited growth at *a*_*w*_ = 0.99. This separation led to huge confidence intervals and infinite parameters in logistic regression. To overcome this issue, the method proposed by Heinze and Schemper (68) was applied. Briefly, this method aims to maximize a penalized likelihood to get a finite estimate of the parameters. It is available in the logistf package.

#### Effect of environmental conditions on mycotoxin production

In some grown replicates, mycotoxin levels were below the detectable limit of quantification, resulting in left-censored observations. Common practices for handling such incomplete data observations involve either discarding values below the quantitation limit or substituting them with an arbitrary value (i.e., half of the quantitation threshold). However, these approaches may (i) discard valuable information from the original data and/or (ii) introduce biases in summary statistical calculations (42, 69).

Therefore, for each species and associated mycotoxin group, a censored version of a two-way ANOVA was done. This version of the ANOVA allows testing differences between groups on a quantitative variable, while retaining left-censored observations. The effect of θ, *a*_*w*_, and their interaction was tested, and the model having the best BIC was selected. The function from the NADA2 package (v.1.1.; [70]) was used.

#### Correlations between mycotoxin production and growth parameters

The correlation between mycotoxin production and growth parameters was investigated using a PCA on the ranks of both growth parameters and mycotoxin quantification, for each species and isolate. To mitigate scale effects resulting from the different mycotoxins, the ranks of mycotoxins (i.e., the position of a mycotoxin concentration within its group, with the lowest value assigned rank 1 and tied values sharing the same rank) were computed within each group of mycotoxins yielded by the *Fusarium* species (TCTB [DON + 15-ADON] for *F. graminearum*, TCTA [DAS + T-2 + HT-2] for *F. poae* and *F. langsethiae,* and ENNs [ENNA + ENNA1 + ENNB + ENNB1] for *F. avenaceum* and *F. tricinctum*). To take into account the fact that under challenging growth conditions, some samples could have not finished their growth at 14 days, the OD at 14 days was included in the analysis when mycotoxins were measured. PCA on the ranks allowed to keep many values without introducing biases caused by arbitrary data substitution.

Data acquisition was performed during a single experimental iteration. The resulting data sets were then processed and statistically analyzed using R Studio software version 4.3.1 (16 June 2023). A level of α = 0.05 was used as the threshold for statistical significance.

## Data Availability

Data and scripts are available in the Data INRAE repository: https://doi.org/10.57745/HXDW3C.

## SUPPLEMENTAL MATERIAL

Supplemental material aem.01832-24-s0001.docx Figures S1 to S5; Tables S1 to S8.

ASM does not own the copyrights to Supplemental Material that may be linked to, or accessed through, an article. The authors have granted ASM a non-exclusive, world-wide license to publish the Supplemental Material files. Please contact the corresponding author directly for reuse.

## References

[1] Xu X-M, Parry DW, Nicholson P, Thomsett MA, Simpson D, Edwards SG, Cooke BM, Doohan FM, Brennan JM, Moretti A, Tocco G, Mule G, Hornok L, Giczey G, Tatnell J. 2005. Predominance and association of pathogenic fungi causing Fusarium ear blightin wheat in four European countries. Eur J Plant Pathol 112:143–154. doi:10.1007/s10658-005-2446-7

[2] Beccari G, Colasante V, Tini F, Senatore MT, Prodi A, Sulyok M, Covarelli L. 2018. Causal agents of Fusarium head blight of durum wheat (Triticum durum Desf.) in central Italy and their in vitro biosynthesis of secondary metabolites. Food Microbiol 70:17–27. doi:10.1016/j.fm.2017.08.016 29173624

[3] Vogelgsang S, Beyer M, Pasquali M, Jenny E, Musa T, Bucheli TD, Wettstein FE, Forrer H-R. 2019. An eight-year survey of wheat shows distinctive effects of cropping factors on different Fusarium species and associated mycotoxins. Eur J Agron 105:62–77. doi:10.1016/j.eja.2019.01.002

[4] Valverde-Bogantes E, Bianchini A, Herr JR, Rose DJ, Wegulo SN, Hallen-Adams HE. 2020. Recent population changes of Fusarium head blight pathogens: drivers and implications. Canadian Journal of Plant Pathology 42:315–329. doi:10.1080/07060661.2019.1680442

[5] Infantino A, Belocchi A, Quaranta F, Reverberi M, Beccaccioli M, Lombardi D, Vitale M. 2023. Effects of climate change on the distribution of Fusarium spp. in Italy. Sci Total Environ 882:163640. doi:10.1016/j.scitotenv.2023.163640 37087011

[6] Dubois C, Roucou A. 2023. L’environnement agroclimatique agit sur L’équilibre de deux espèces. Perspectives agricoles.

[7] Osborne LE, Stein JM. 2007. Epidemiology of Fusarium head blight on small-grain cereals. Int J Food Microbiol 119:103–108. doi:10.1016/j.ijfoodmicro.2007.07.032 17716761

[8] van der Lee T, Zhang H, van Diepeningen A, Waalwijk C. 2015. Biogeography of Fusarium graminearum species complex and chemotypes: a review. Food Addit Contam Part A Chem Anal Control Expo Risk Assess 32:453–460. doi:10.1080/19440049.2014.984244 25530109 PMC4376211

[9] Kokkonen M, Medina A, Magan N. 2012. Comparative study of water and temperature relations of growth and T-2/HT-2 toxin production by strains of Fusarium sporotrichioides and Fusarium langsethiae. WMJ 5:365–372. doi:10.3920/WMJ2012.1406

[10] Dinolfo MI, Stenglein S. 2014. Fusarium poae and mycotoxins: potential risk for consumers. Bol Soc Argent Bot 49:5–20. doi:10.31055/1851.2372.v49.n1.7786

[11] Gautier C, Pinson-Gadais L, Richard-Forget F. 2020. Fusarium mycotoxins enniatins: an updated review of their occurrence, the producing Fusarium species, and the abiotic determinants of their accumulation in crop harvests. J Agric Food Chem 68:4788–4798. doi:10.1021/acs.jafc.0c00411 32243758

[12] Langseth W, Bernhoft A, Rundberget T, Kosiak B, Gareis M. 1998. Mycotoxin production and cytotoxicity of Fusarium strains isolated from norwegian cereals. Mycopathologia 144:103–113. doi:10.1023/a:1007016820879 10481290

[13] Cundliffe E, Cannon M, Davies J. 1974. Mechanism of inhibition of eukaryotic protein synthesis by trichothecene fungal toxins. Proc Natl Acad Sci USA 71:30–34. doi:10.1073/pnas.71.1.30 4521056 PMC387925

[14] Ueno Y, Matsumoto H. 1975. Inactivation of some thiol-enzymes by trichothecene mycotoxins from Fusarium species. Chem Pharm Bull 23:2439–2442. doi:10.1248/cpb.23.2439 1212759

[15] Cundliffe E, Davies JE. 1977. Inhibition of initiation, elongation, and termination of eukaryotic protein synthesis by trichothecene fungal toxins. Antimicrob Agents Chemother 11:491–499. doi:10.1128/AAC.11.3.491 856003 PMC352012

[16] Eudes F, Comeau A, Rioux S, Collin J. 2000. Phytotoxicité de huit mycotoxines associées à la fusariose de l’épi chez le blé. Canadian Journal of Plant Pathology 22:286–292. doi:10.1080/07060660009500477

[17] Steinmetz WE, Rodarte CB, Lin A. 2009. 3D QSAR study of the toxicity of trichothecene mycotoxins. Eur J Med Chem 44:4485–4489. doi:10.1016/j.ejmech.2009.06.012 19586689

[18] Knutsen HK, Alexander J, Barregård L, Bignami M, Brüschweiler B, Ceccatelli S, Cottrill B, Dinovi M, Grasl-Kraupp B, Hogstrand C, et al.. 2017. Risks to human and animal health related to the presence of deoxynivalenol and its acetylated and modified forms in food and feed. EFSA J 15:e04718. doi:10.2903/j.efsa.2017.4718 32625635 PMC7010102

[19] Fink-Gremmels J, Malekinejad H. 2007. Clinical effects and biochemical mechanisms associated with exposure to the mycoestrogen zearalenone. Animal Feed Science and Technology 137:326–341. doi:10.1016/j.anifeedsci.2007.06.008

[20] Mallebrera B, Prosperini A, Font G, Ruiz MJ. 2018. In vitro mechanisms of beauvericin toxicity: a review. Food Chem Toxicol 111:537–545. doi:10.1016/j.fct.2017.11.019 29154952

[21] Pinotti L, Ottoboni M, Giromini C, Dell’Orto V, Cheli F. 2016. Mycotoxin contamination in the EU feed supply chain: a focus on cereal byproducts. Toxins (Basel) 8:45. doi:10.3390/toxins8020045 26891326 PMC4773798

[22] Garcia-Cela E, Verheecke-Vaessen C, Magan N, Medina A. 2018. The ``omics’’ contributions to the understanding of mycotoxin production under diverse environmental conditions. Curr Opin Food Sci 23:97–104. doi:10.1016/j.cofs.2018.08.005

[23] Kos J, Anić M, Radić B, Zadravec M, Janić Hajnal E, Pleadin J. 2023. Climate change-a global threat resulting in increasing mycotoxin occurrence. Foods 12:2704. doi:10.3390/foods12142704 37509796 PMC10379110

[24] Beyer M, Pogoda F, Pallez M, Lazic J, Hoffmann L, Pasquali M. 2014. Evidence for a reversible drought induced shift in the species composition of mycotoxin producing Fusarium head blight pathogens isolated from symptomatic wheat heads. Int J Food Microbiol 182–183:51–56. doi:10.1016/j.ijfoodmicro.2014.05.002 24859190

[25] Covarelli L, Beccari G, Prodi A, Generotti S, Etruschi F, Juan C, Ferrer E, Mañes J. 2015. Fusarium species, chemotype characterisation and trichothecene contamination of durum and soft wheat in an area of central Italy. J Sci Food Agric 95:540–551. doi:10.1002/jsfa.6772 24909776

[26] Banik M, Beyene M, Wang X. 2018. Fusarium head blight of barley in manitoba. The Canadian Phytopathological Society

[27] Nogueira MS, Decundo J, Martinez M, Dieguez SN, Moreyra F, Moreno MV, Stenglein SA. 2018. Natural contamination with mycotoxins produced by Fusarium graminearum and Fusarium poae in malting barley in argentina. Toxins (Basel) 10:78. doi:10.3390/toxins10020078 29439459 PMC5848179

[28] Fox EM, Howlett BJ. 2008. Secondary metabolism: regulation and role in fungal biology. Curr Opin Microbiol 11:481–487. doi:10.1016/j.mib.2008.10.007 18973828

[29] Audenaert K, Vanheule A, Höfte M, Haesaert G. 2013. Deoxynivalenol: a major player in the multifaceted response of Fusarium to its environment. Toxins (Basel) 6:1–19. doi:10.3390/toxins6010001 24451843 PMC3920246

[30] Hope R, Aldred D, Magan N. 2005. Comparison of environmental profiles for growth and deoxynivalenol production by Fusarium culmorum and F. graminearum on wheat grain. Lett Appl Microbiol 40:295–300. doi:10.1111/j.1472-765X.2005.01674.x 15752221

[31] Nazari L, Pattori E, Terzi V, Morcia C, Rossi V. 2014. Influence of temperature on infection, growth, and mycotoxin production by Fusarium langsethiae and F. sporotrichioides in durum wheat. Food Microbiol 39:19–26. doi:10.1016/j.fm.2013.10.009 24387848

[32] Verheecke-Vaessen C, Lopez-Pietro A, Garcia-Cela E, Medina A, Magan N. 2022. Intra-species variability in Fusarium langsethiae strains in growth and T-2/HT-2 mycotoxin production in response to climate change abiotic factors. WMJ 15:27–34. doi:10.3920/WMJ2020.2584

[33] Perrone G, Ferrara M, Medina A, Pascale M, Magan N. 2020. Toxigenic fungi and mycotoxins in a climate change scenario: ecology, genomics, distribution, prediction and prevention of the risk. Microorganisms 8:1496. doi:10.3390/microorganisms8101496 33003323 PMC7601308

[34] Medina A, Magan N. 2010. Comparisons of water activity and temperature impacts on growth of Fusarium langsethiae strains from northern Europe on oat-based media. Int J Food Microbiol 142:365–369. doi:10.1016/j.ijfoodmicro.2010.07.021 20688410

[35] Peter Mshelia L, Selamat J, Iskandar Putra Samsudin N, Rafii MY, Abdul Mutalib N-A, Nordin N, Berthiller F. 2020. Effect of temperature, water activity and carbon dioxide on fungal growth and mycotoxin production of acclimatised isolates of Fusarium verticillioides and F. graminearum. Toxins (Basel) 12:478. doi:10.3390/toxins12080478 32731333 PMC7472189

[36] Yu S, Jia B, Li K, Zhou H, Lai W, Tang Y, Yan Z, Sun W, Liu N, Yu D, Wu A. 2021. Pre-warning of abiotic factors in maize required for potential contamination of Fusarium mycotoxins via response surface analysis. Food Control 121:107570. doi:10.1016/j.foodcont.2020.107570

[37] Boixel A-L, Delestre G, Legeay J, Chelle M, Suffert F. 2019. Phenotyping thermal responses of yeasts and yeast-like microorganisms at the individual and population levels: proof-of-concept, development and application of an experimental framework to a plant pathogen. Microb Ecol 78:42–56. doi:10.1007/s00248-018-1253-6 30280234

[38] Hameed T, Motsi N, Bignell E, Tanaka RJ. 2024. Inferring fungal growth rates from optical density data. PLoS Comput Biol 20:e1012105. doi:10.1371/journal.pcbi.1012105 38753887 PMC11098479

[39] Heidorn PB. 2008. Shedding light on the dark data in the long tail of science. lib 57:280–299. doi:10.1353/lib.0.0036

[40] Zamir D. 2013. Where have all the crop phenotypes gone? PLoS Biol 11:e1001595. doi:10.1371/journal.pbio.1001595 23824246 PMC3692434

[41] Wood SN. 2017. Generalized Additive Models: an introduction with R SECOND EDITION. CRC Press.

[42] Shoari N, Dubé J-S. 2017. Toward improved analysis of concentration data: embracing nondetects. Environ Toxicol Chem 37:643–656. doi:10.1002/etc.4046 29168890

[43] Manstretta V, Morcia C, Terzi V, Rossi V. 2016. Germination of Fusarium graminearum ascospores and wheat infection are affected by dry periods and by temperature and humidity during dry periods. Phytopathology 106:262–269. doi:10.1094/PHYTO-05-15-0118-R 26623994

[44] Kokkonen M, Ojala L, Parikka P, Jestoi M. 2010. Mycotoxin production of selected Fusarium species at different culture conditions. Int J Food Microbiol 143:17–25. doi:10.1016/j.ijfoodmicro.2010.07.015 20708288

[45] Medina A, Magan N. 2011. Temperature and water activity effects on production of T-2 and HT-2 by Fusarium langsethiae strains from north European countries. Food Microbiol 28:392–398. doi:10.1016/j.fm.2010.09.012 21356443

[46] Verheecke-Vaessen C, Garcia-Cela E, Lopez-Prieto A, Osk Jonsdottir I, Medina A, Magan N. 2021. Water and temperature relations of Fusarium langsethiae strains and modelling of growth and T-2 and HT-2 mycotoxin production on oat-based matrices. Int J Food Microbiol 348:109203. doi:10.1016/j.ijfoodmicro.2021.109203 33930835

[47] Torp M, Nirenberg HI. 2004. Fusarium langsethiae sp. nov. on cereals in Europe. Int J Food Microbiol 95:247–256. doi:10.1016/j.ijfoodmicro.2003.12.014 15337590

[48] Imathiu SM, Hare MC, Ray RV, Back M, Edwards SG. 2010. Evaluation of pathogenicity and aggressiveness of F. langsethiae on oat and wheat seedlings relative to known seedling blight pathogens. Eur J Plant Pathol 126:203–216. doi:10.1007/s10658-009-9533-0

[49] Hjelkrem A-GR, Aamot HU, Brodal G, Strand EC, Torp T, Edwards SG, Dill-Macky R, Hofgaard IS. 2018. HT-2 and T-2 toxins in norwegian oat grains related to weather conditions at different growth stages. Eur J Plant Pathol 151:501–514. doi:10.1007/s10658-017-1394-3

[50] Zonno MC, Vurro M. 2002. Inhibition of germination of Orobanche ramosa seeds by Fusariumu toxins. Phytoparasitica 30:519–524. doi:10.1007/BF02979757

[51] Ma L-J, Geiser DM, Proctor RH, Rooney AP, O’Donnell K, Trail F, Gardiner DM, Manners JM, Kazan K. 2013. Fusarium pathogenomics. Annu Rev Microbiol 67:399–416. doi:10.1146/annurev-micro-092412-155650 24024636

[52] Parikka P, Hakala K, Tiilikkala K. 2012. Expected shifts in Fusarium species’ composition on cereal grain in Northern Europe due to climatic change. Food Additives & Contaminants: Part A 29:1543–1555. doi:10.1080/19440049.2012.680613 22554046

[53] Moretti A, Pascale M, Logrieco AF. 2019. Mycotoxin risks under a climate change scenario in Europe. Trends Food Sci Technol 84:38–40. doi:10.1016/j.tifs.2018.03.008

[54] Hay WT, McCormick SP, Vaughan MM. 2021. Effects of atmospheric CO_2_ and temperature on wheat and corn susceptibility to Fusarium graminearum and deoxynivalenol contamination. Plants (Basel) 10:2582. doi:10.3390/plants10122582 34961056 PMC8709488

[55] Kahla A, Verheecke-Vaessen C, Delpino-Deelias M, Gutierrez-Pozo M, Medina A, Magan N, Doohan F. 2023. Acclimatisation of Fusarium langsethiae, F. poae and F. sporotrichioides to elevated CO_2_: Impact on fungal growth and mycotoxin production on oat-based media. Int J Food Microbiol 394:110176. doi:10.1016/j.ijfoodmicro.2023.110176 36989929

[56] Bakker MG, Whitaker BK, McCormick SP, Ainsworth EA, Vaughan MM. 2023. Manipulating atmospheric CO_2_ concentration induces shifts in wheat leaf and spike microbiomes and in Fusarium pathogen communities. Front Microbiol 14:1271219. doi:10.3389/fmicb.2023.1271219 37881249 PMC10595150

[57] Ponts N, Couedelo L, Pinson-Gadais L, Verdal-Bonnin M-N, Barreau C, Richard-Forget F. 2009. Fusarium response to oxidative stress by H2O2 is trichothecene chemotype-dependent. FEMS Microbiol Lett 293:255–262. doi:10.1111/j.1574-6968.2009.01521.x 19239497

[58] Battilani P, Camardo Leggieri M, Rossi V, Giorni P. 2013. AFLA-maize, a mechanistic model for aspergillus flavus infection and aflatoxin B1 contamination in maize. Comput Electron Agric 94:38–46. doi:10.1016/j.compag.2013.03.005

[59] van der Fels-Klerx HJ, Olesen JE, Madsen MS, Goedhart PW. 2012. Climate change increases deoxynivalenol contamination of wheat in north-western Europe. Food Additives & Contaminants: Part A 29:1593–1604. doi:10.1080/19440049.2012.691555 22742589

[60] Arseniuk E, Foremska E, G T, Óral G, Chełkowski J. 1999. Fusarium head blight reactions and accumulation of deoxynivalenol (DON) and some of its derivatives in kernels of wheat, triticale and rye. Journal of Phytopathology 147:577–590. doi:10.1046/j.1439-0434.1999.00433.x

[61] Karlsson I, Persson P, Friberg H. 2021. Fusarium head blight from a microbiome perspective. Front Microbiol 12:628373. doi:10.3389/fmicb.2021.628373 33732223 PMC7956947

[62] Boutigny A-L, Gautier A, Basler R, Dauthieux F, Leite S, Valade R, Aguayo J, Ioos R, Laval V. 2019. Metabarcoding targeting the EF1 alpha region to assess Fusarium diversity on cereals. PLoS ONE 14:e0207988. doi:10.1371/journal.pone.0207988 30633747 PMC6329491

[63] Siou D, Gélisse S, Laval V, Elbelt S, Repinçay C, Bourdat-Deschamps M, Suffert F, Lannou C. 2015. Interactions between head blight pathogens: consequences for disease development and toxin production in wheat spikes. Appl Environ Microbiol 81:957–965. doi:10.1128/AEM.02879-14 25416772 PMC4292474

[64] Baeza R, Pérez A, Sánchez V, Zamora MC, Chirife J. 2010. Evaluation of norrish’s equation for correlating the water activity of highly concentrated solutions of sugars, polyols, and polyethylene glycols. Food Bioprocess Technol 3:87–92. doi:10.1007/s11947-007-0052-8

[65] Ritz C, Baty F, Streibig JC, Gerhard D. 2015. Dose-response analysis using R. PLoS One 10:e0146021. doi:10.1371/journal.pone.0146021 26717316 PMC4696819

[66] Taudiere A, Violle C. 2016. Cati: an R package using functional traits to detect and quantify multi‐level community assembly processes. Ecography 39:699–708. doi:10.1111/ecog.01433

[67] Albert A, Anderson JA. 1984. On the existence of maximum likelihood estimates in logistic regression models. Biometrika 71:1–10. doi:10.1093/biomet/71.1.1

[68] Heinze G, Schemper M. 2002. A solution to the problem of separation in logistic regression. Stat Med 21:2409–2419. doi:10.1002/sim.1047 12210625

[69] Shoari N, Dubé J-S, Chenouri S. 2016. On the use of the substitution method in left-censored environmental data. Hum Ecol Risk Assess 22:435–446. doi:10.1080/10807039.2015.1079481

[70] Julian P, Helsel D. 2024. NADA2: data analysis for censored environmental data

