# Supplementary material for: Ecophysiological behavior of major Fusarium species in response to combinations of temperature and water activity constraints
Source: Appl Environ Microbiol. 2025 Jun 10;91(7):e01832-24. doi: 10.1128/aem.01832-24 (PMC12285238; doi:10.1128/aem.01832-24)
Supplement: Supplemental material — Figures S1 to S5; Tables S1 to S8. [file aem.01832-24-s0001.docx]

# **Ecophysiological behaviour of major *Fusarium* species in response to combinations of temperature and water activity constraints**

Marie-Anne Garcia^a*^, Rémi Mahmoud^bc*^, Marie-Odile Bancal^b^, Pierre Bancal^b^, Stéphane Bernillon^a^, Laetitia Pinson-Gadais^a^, Florence Richard-Forget^a^, Marie Foulongne-Oriol^a^#

^a^ INRAE, MYCSA, 33882 Villenave d’Ornon, France

^b^ Université Paris-Saclay, INRAE, AgroParisTech, EcoSys, 91120 Palaiseau, France

^c^ Institut Agro, Univ Rennes, CNRS, IRMAR-UMR 6625, 35042 Rennes, France

*M.A.G. and R.M. contributed equally to this work.

# Corresponding author : address correspondence to Marie Foulongne-Oriol, [marie.foulongne-oriol@inrae.fr](mailto:marie.foulongne-oriol@inrae.fr)

Mailing address : INRAE, UR1264 Mycologie et Sécurité des Aliments, bâtiment Qualis, 71 avenue Edouard Bourlaux, CS 20032, F-33882 Villenave d’Ornon cedex, France

Running title : *Fusarium* spp. behaviours under abiotic constraints

Supplemental material


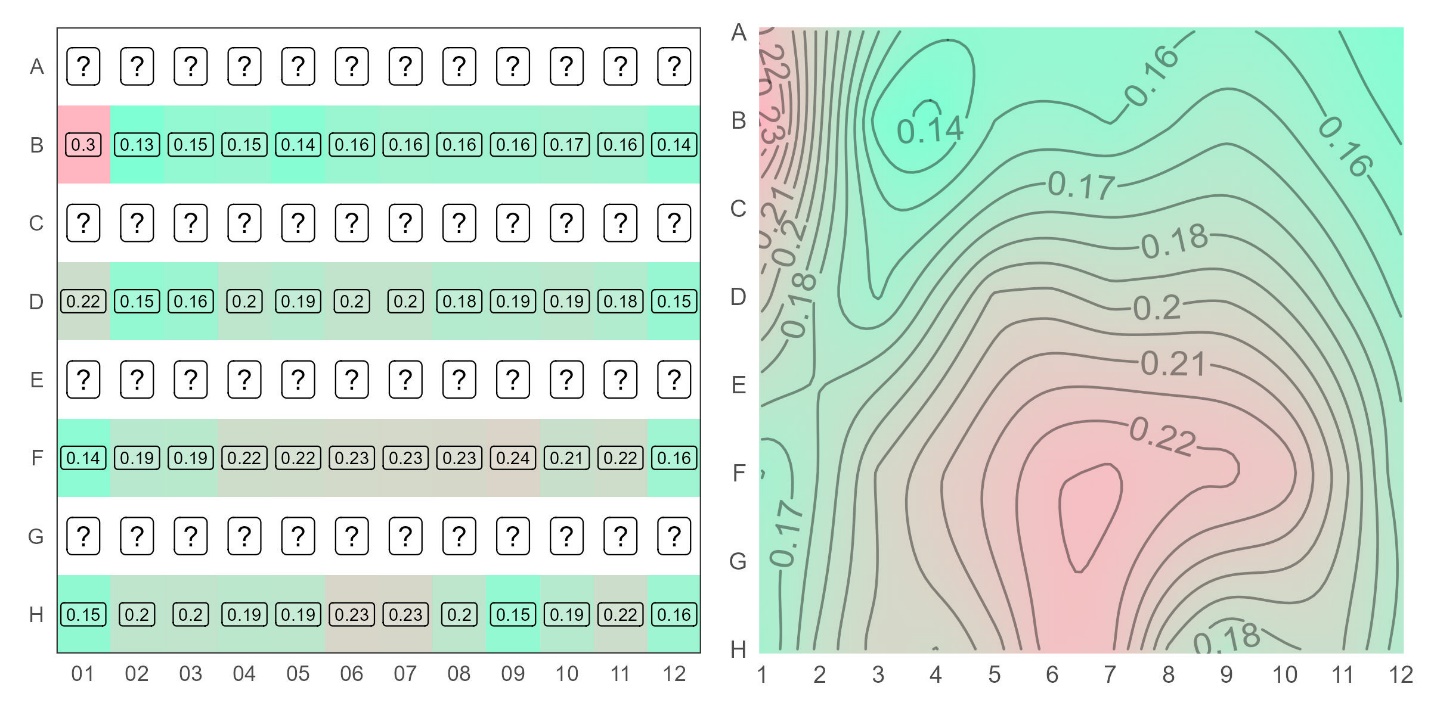


**Supplementary Figure 1: Spatial distribution of plate and medium absorbance at λ = 630nm.** Fungal replicates were inoculated on lines A, C, E and G and blanks (MS at the correct a_w_) were placed in lines B, D, F and H of each plate (left part of the figure). Growth measurements were done on inoculated wells (indicated by ? symbol) and on non-inoculated wells (example of values in lines B, D, F and H). Blank absorption was modelled for each plate (right part of the figure), using a spatial Generalised Additive Model (GAM) to interpolate the optical density (OD) values across the entire plate.


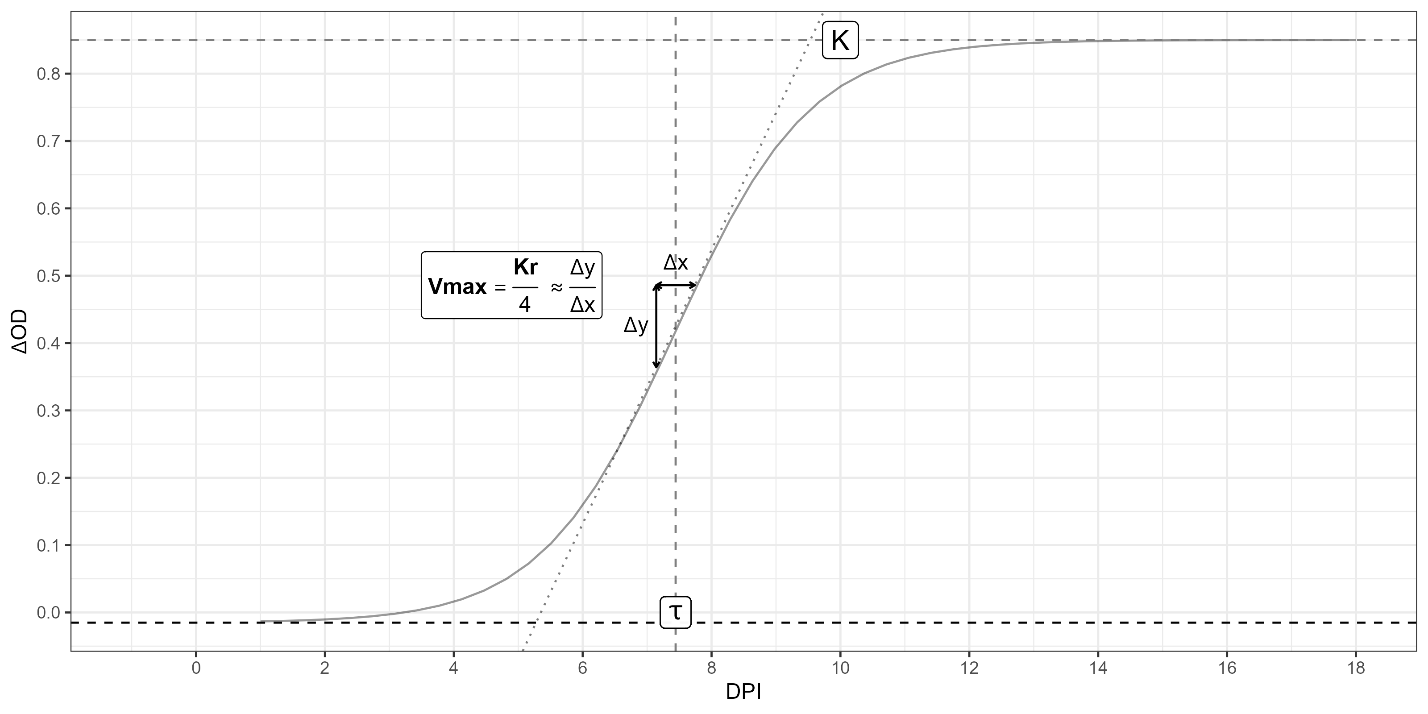


**Supplementary Figure 2 : Graphical significance of growth parameters (𝜏, K, r and Vmax).** Growth parameters were estimated from fungal growth curves for six replicates of the five *Fusarium* species at four ϴ (15, 20, 25 and 30°C) and six a_w_ (0.94, 0.95, 0.96, 0.97, 0.98 and 0.99). K represents the carrying coefficient of the environment and it is expressed without unit, r is the relative growth rate in OD_unit_.days^-1^ and τ is the time of the inflection point of the curve expressed in days. The growth rate at inflexion time (Vmax expressed in OD_unit_.days^-1^) is obtained as rK/4.


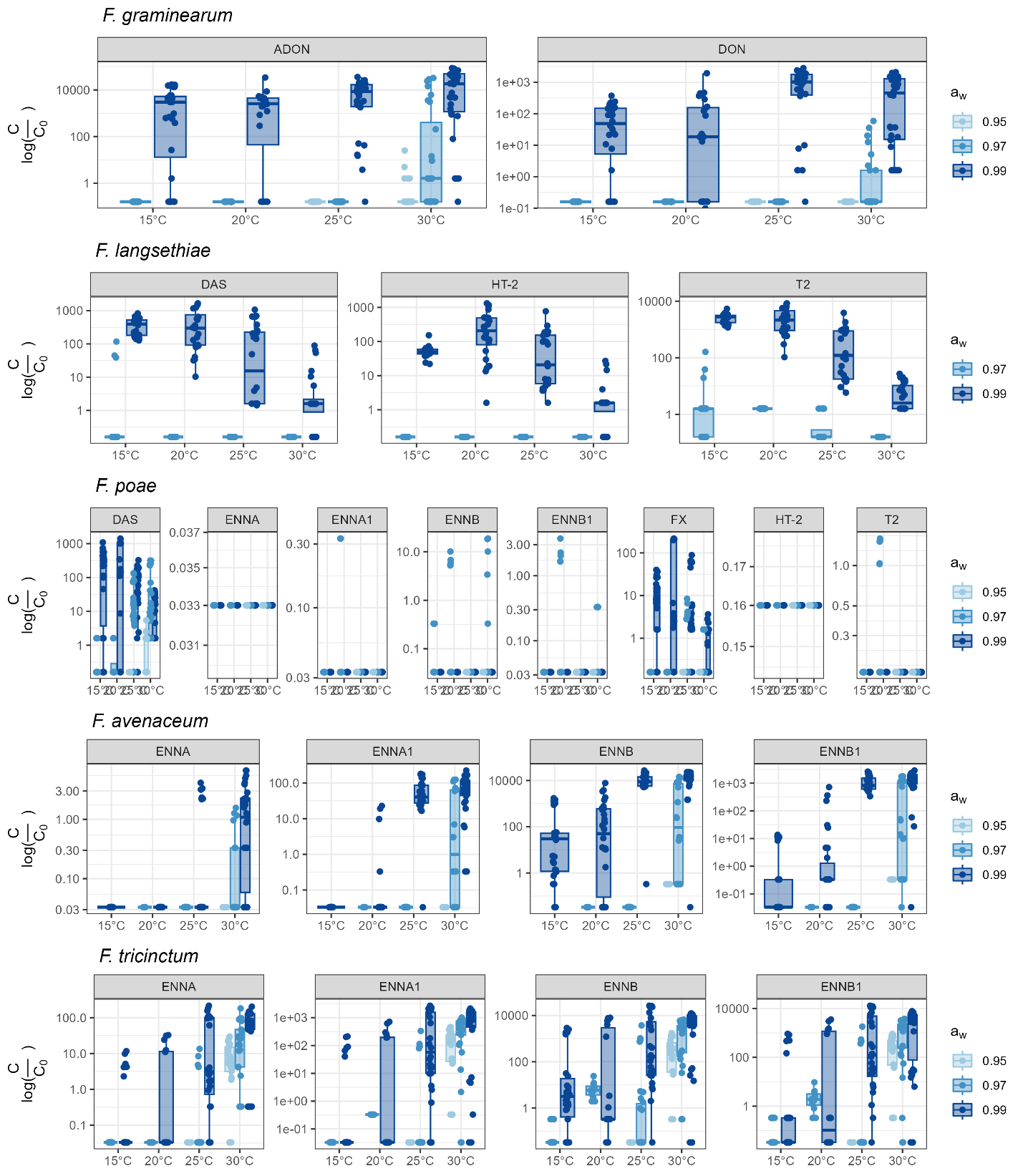


**Supplementary Figure 3 : Details of mycotoxin produced by the five *Fusarium* species under environmental variation.** Mycotoxins produced (log-transformed, y-axis) by *F. graminearum*, *F. langsethiae*, *F. poae, F. avenaceum* and *F. tricinctum* at four ϴ (15, 20, 25 and 30°C, x-axis) and three a_w_ levels (0.95, 0.97 and 0.99). Each dot represents one replicate and the boxplots show the intraspecific diversity in terms of mycotoxin production within each *Fusarium* species.


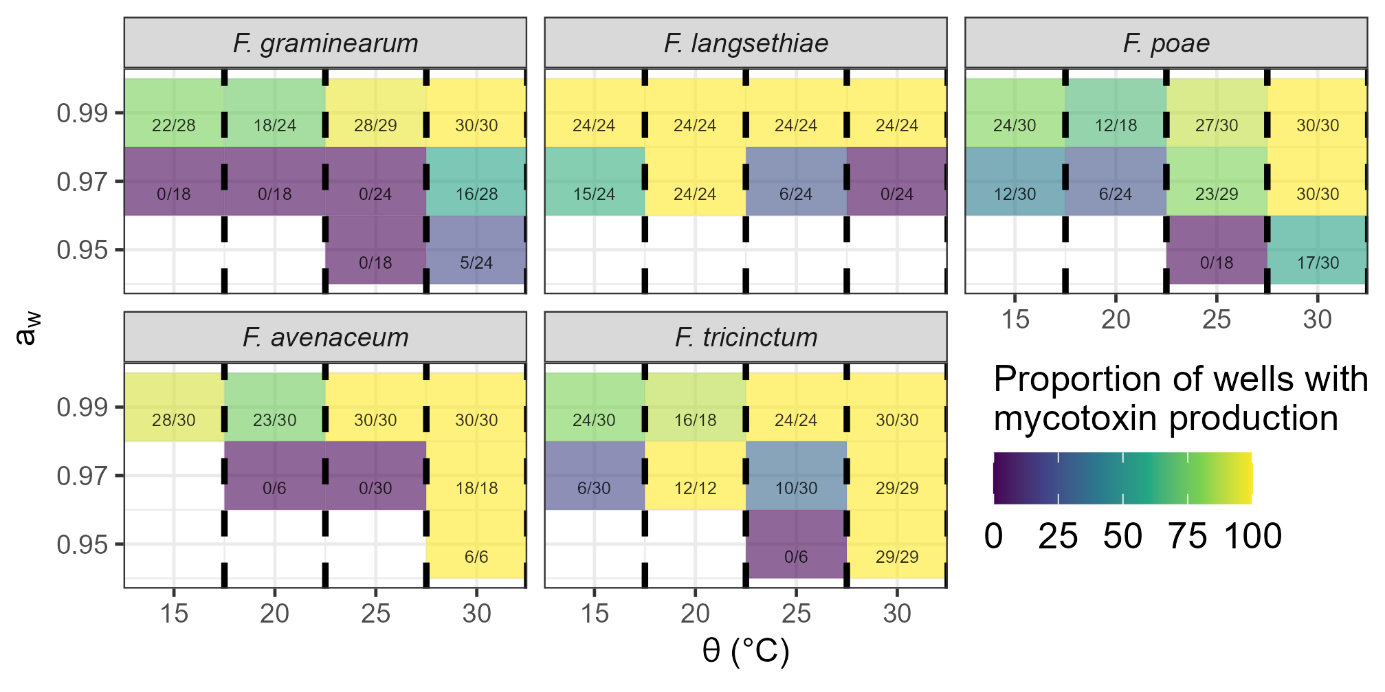


**Supplementary Figure 4 : Frequency of mycotoxin production for the five *Fusarium* species depending on environmental variation.** The number of replicates in which mycotoxins were detected is represented on the total number of grown replicates for *F. graminearum, F. langsethiae, F. poae, F. avenaceum* and *F. tricinctum* for four ϴ (15, 20, 25 and 30°C, x-axis) and three a_w_ levels (0.95, 0.97 and 0.99, y-axis). Missing data corresponds to wells where no growth was observed.


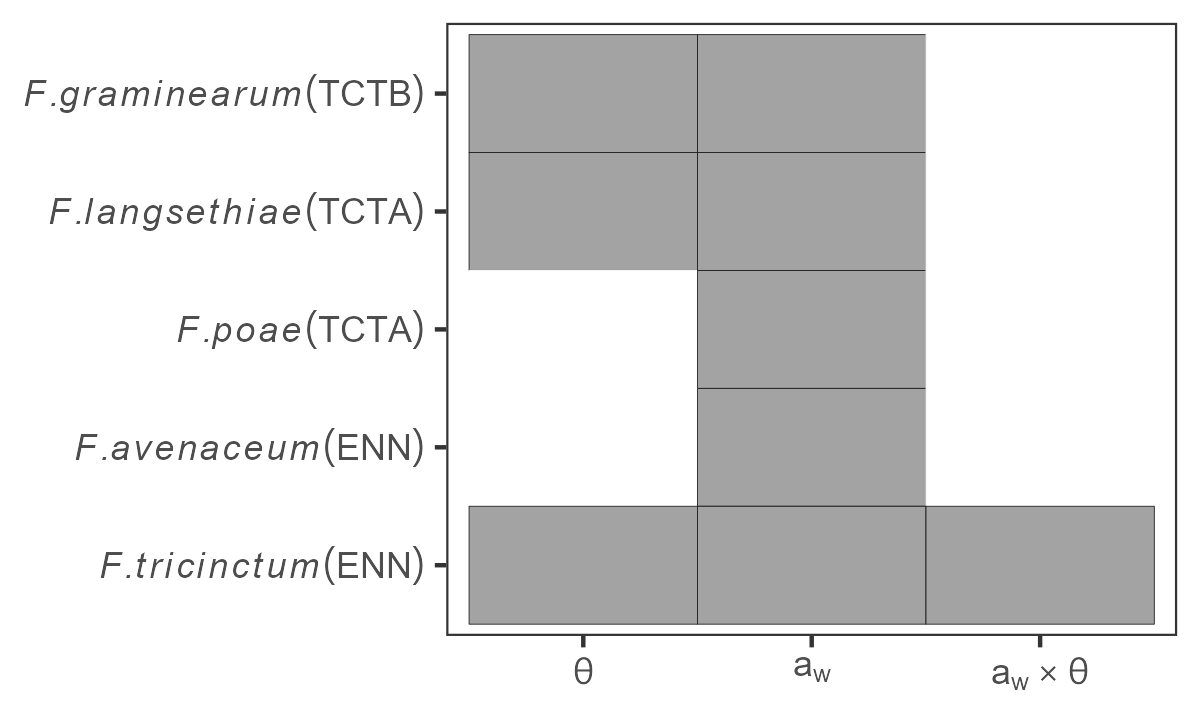


**Supplementary Figure 5 : Effects of environmental factors (a_w_ and ϴ) and their interaction (a_w_ × ϴ) on mycotoxin production for the five *Fusarium* species.** Grey tiles indicate the significant effect (retained in the model with the best Bayesian Information Criterion) of environmental factors (a_w_ and ϴ) and their interaction (x-axis) on mycotoxins produced by *F. graminearum*, *F. langsethiae*, *F. poae, F.* *avenaceum* and *F. tricinctum* (y-axis).

**Supplementary Table 1 : ANOVA results of interspecific diversity of the growth parameters (𝜏, K, r and Vmax).** The value of Fisher’s coefficient (F), the p-value (Pr(>F)) and the proportion of variance explained (% Var) by the effect of species, environmental factors (a_w_ and ϴ) and their interactions (a_w_ × ϴ, Species × a_w_ and Species × ϴ) are reported for each growth parameters. Pr(>F) highlighted in bold indicates the significance of the Fisher test.

|  | τ | | | K | | | r | | | Vmax | | |
| --- | --- | --- | --- | --- | --- | --- | --- | --- | --- | --- | --- | --- |
| Factor | F | Pr(>F) | % Var | F | Pr(>F) | % Var | F | Pr(>F) | % Var | F | Pr(>F) | % Var |
| Species | 1.14 | 0.34 | 0.53 | 3.79 | **<0.05** | 4.24 | 1.57 | 0.19 | 1.46 | 0.47 | 0.75 | 0.84 |
| a_w_ | 24.02 | **<0.05** | 11.22 | 35.80 | **<0.05** | 40.03 | 6.69 | **<0.05** | 6.25 | 5.34 | **<0.05** | 9.46 |
| ϴ | 85.84 | **<0.05** | 40.10 | 1.34 | 0.27 | 1.50 | 27.62 | **<0.05** | 25.78 | 2.97 | **<0.05** | 5.26 |
| a_w_ × ϴ | 73.94 | **<0.05** | 34.54 | 22.06 | **<0.05** | 24.66 | 8.13 | **<0.05** | 7.59 | 26.09 | **<0.05** | 46.25 |
| Species × a_w_ | 3.16 | **<0.05** | 1.47 | 3.32 | **<0.05** | 3.72 | 2.18 | **<0.05** | 2.04 | 3.22 | **<0.05** | 5.70 |
| Species × ϴ | 2.46 | **<0.05** | 1.15 | 2.50 | **<0.05** | 2.80 | 4.95 | **<0.05** | 4.62 | 3.18 | **<0.05** | 5.63 |
| Residual |  |  | 10.98 |  |  | 23.06 |  |  | 52.26 |  |  | 26.85 |

**Supplementary Table 2 : ANOVA results of the *F. graminearum* growth parameters (𝜏, K, r and Vmax).** The value of Fisher’s coefficient (F), the p-value (Pr(>F)) and the proportion of variance explained (% Var) by the effect of isolate, environmental factors (a_w_ and ϴ) and their interactions (a_w_ × ϴ, Isolate × a_w_ and Isolate × ϴ) are reported for each growth parameters of *F. graminearum*. Pr(>F) highlighted in bold indicates the significance of the Fisher test.

|  | τ | | | K | | | r | | | Vmax | | |
| --- | --- | --- | --- | --- | --- | --- | --- | --- | --- | --- | --- | --- |
| Factor | F | Pr(>F) | % Var | F | Pr(>F) | % Var | F | Pr(>F) | % Var | F | Pr(>F) | % Var |
| Isolate | 26.68 | **<0.05** | 1.66 | 37.02 | **<0.05** | 15.84 | 3.91 | **<0.05** | 10.03 | 49.58 | **<0.05** | 24.55 |
| a_w_ | 242.42 | **<0.05** | 15.09 | 111.42 | **<0.05** | 47.68 | 2.44 | **<0.05** | 6.25 | 77.99 | **<0.05** | 38.63 |
| ϴ | 1,255.53 | **<0.05** | 78.13 | 65.66 | **<0.05** | 28.10 | 17.59 | **<0.05** | 45.11 | 41.17 | **<0.05** | 20.39 |
| a_w_ × ϴ | 75.80 | **<0.05** | 4.72 | 5.67 | **<0.05** | 2.43 | 2.17 | **<0.05** | 5.56 | 4.44 | **<0.05** | 2.20 |
| Isolate × a_w_ | 2.17 | **<0.05** | 0.13 | 3.48 | **<0.05** | 1.49 | 7.36 | **<0.05** | 18.88 | 10.73 | **<0.05** | 5.31 |
| Isolate × ϴ | 3.29 | **<0.05** | 0.20 | 9.42 | **<0.05** | 4.03 | 4.52 | **<0.05** | 11.59 | 16.99 | **<0.05** | 8.42 |
| Residual |  |  | 0.06 |  |  | 0.43 |  |  | 2.56 |  |  | 0.50 |

**Supplementary Table 3 : ANOVA results of the *F. langsethiae* growth parameter (𝜏, K, r and Vmax).** The value of Fisher’s coefficient (F), the p-value (Pr(>F)) and the proportion of variance explained (% Var) by the effect of isolate, environmental factors (a_w_ and ϴ) and their interactions (a_w_ × ϴ, Isolate × a_w_ and Isolate × ϴ) are reported for each growth parameters of *F. langsethiae*. Pr(>F) highlighted in bold indicates the significance of the Fisher test.

|  | τ | | | K | | | r | | | Vmax | | |
| --- | --- | --- | --- | --- | --- | --- | --- | --- | --- | --- | --- | --- |
| Factor | F | Pr(>F) | % Var | F | Pr(>F) | % Var | F | Pr(>F) | % Var | F | Pr(>F) | % Var |
| Isolate | 21.49 | **<0.05** | 5.13 | 27.35 | **<0.05** | 14.80 | 1.79 | 0.13 | 1.84 | 13.49 | **<0.05** | 8.33 |
| a_w_ | 166.08 | **<0.05** | 39.69 | 108.02 | **<0.05** | 58.44 | 15.14 | **<0.05** | 15.62 | 48.11 | **<0.05** | 29.70 |
| ϴ | 172.47 | **<0.05** | 41.22 | 7.86 | **<0.05** | 4.25 | 56.39 | **<0.05** | 58.16 | 70.07 | **<0.05** | 43.25 |
| a_w_ × ϴ | 10.53 | **<0.05** | 2.52 | 15.02 | **<0.05** | 8.13 | 14.22 | **<0.05** | 14.67 | 13.06 | **<0.05** | 8.06 |
| Isolate × a_w_ | 13.21 | **<0.05** | 3.16 | 16.70 | **<0.05** | 9.04 | 5.00 | **<0.05** | 5.16 | 7.20 | **<0.05** | 4.44 |
| Isolate × ϴ | 33.68 | **<0.05** | 8.05 | 8.89 | **<0.05** | 4.81 | 3.41 | **<0.05** | 3.52 | 9.07 | **<0.05** | 5.60 |
| Residual |  |  | 0.24 |  |  | 0.54 |  |  | 1.03 |  |  | 0.62 |

**Supplementary Table 4 : ANOVA results of the *F. poae* growth parameters (𝜏, K, r and Vmax).** The value of Fisher’s coefficient (F), the p-value (Pr(>F)) and the proportion of variance explained (% Var) by the effect of isolate, environmental factors (a_w_ and ϴ) and their interactions (a_w_ × ϴ, Isolate × a_w_ and Isolate × ϴ) are reported for each growth parameters of *F. poae*. Pr(>F) highlighted in bold indicates the significance of the Fisher test.

|  | τ | | | K | | | r | | | Vmax | | |
| --- | --- | --- | --- | --- | --- | --- | --- | --- | --- | --- | --- | --- |
| Factor | F | Pr(>F) | % Var | F | Pr(>F) | % Var | F | Pr(>F) | % Var | F | Pr(>F) | % Var |
| Isolate | 457.00 | **<0.05** | 14.02 | 28.93 | **<0.05** | 5.43 | 18.58 | **<0.05** | 11.53 | 9.45 | **<0.05** | 3.83 |
| a_w_ | 545.88 | **<0.05** | 16.75 | 317.21 | **<0.05** | 59.51 | 15.72 | **<0.05** | 9.76 | 75.12 | **<0.05** | 30.45 |
| ϴ | 2,131.02 | **<0.05** | 65.39 | 142.15 | **<0.05** | 26.67 | 87.85 | **<0.05** | 54.54 | 119.61 | **<0.05** | 48.48 |
| a_w_ × ϴ | 60.78 | **<0.05** | 1.87 | 19.20 | **<0.05** | 3.60 | 18.01 | **<0.05** | 11.18 | 29.63 | **<0.05** | 12.01 |
| Isolate × a_w_ | 14.39 | **<0.05** | 0.44 | 16.97 | **<0.05** | 3.18 | 13.62 | **<0.05** | 8.46 | 7.12 | **<0.05** | 2.89 |
| Isolate × ϴ | 48.75 | **<0.05** | 1.50 | 7.61 | **<0.05** | 1.43 | 6.29 | **<0.05** | 3.90 | 4.77 | **<0.05** | 1.94 |
| Residual |  |  | 0.03 |  |  | 0.19 |  |  | 0.62 |  |  | 0.41 |

**Supplementary Table 5 : ANOVA results of the *F. avenaceum* growth parameters (𝜏, K, r and Vmax).** The value of Fisher’s coefficient (F), the p-value (Pr(>F)) and the proportion of variance explained (% Var) by the effect of isolate, environmental factors (a_w_ and ϴ) and their interactions (a_w_ × ϴ, Isolate × a_w_ and Isolate × ϴ) are reported for each growth parameters of *F. avenaceum*. Pr(>F) highlighted in bold indicates the significance of the Fisher test.

|  | τ | | | K | | | r | | | Vmax | | |
| --- | --- | --- | --- | --- | --- | --- | --- | --- | --- | --- | --- | --- |
| Factor | F | Pr(>F) | % Var | F | Pr(>F) | % Var | F | Pr(>F) | % Var | F | Pr(>F) | % Var |
| Isolate | 8.80 | **<0.05** | 1.36 | 2.13 | 0.08 | 0.31 | 1.34 | 0.25 | 1.71 | 3.50 | **<0.05** | 1.71 |
| a_w_ | 45.97 | **<0.05** | 7.13 | 620.43 | **<0.05** | 89.20 | 22.04 | **<0.05** | 28.16 | 100.91 | **<0.05** | 49.22 |
| ϴ | 536.94 | **<0.05** | 83.24 | 20.56 | **<0.05** | 2.96 | 41.70 | **<0.05** | 53.27 | 64.96 | **<0.05** | 31.69 |
| a_w_ × ϴ | 22.91 | **<0.05** | 3.55 | 24.83 | **<0.05** | 3.57 | 4.79 | **<0.05** | 6.12 | 20.85 | **<0.05** | 10.17 |
| Isolate × a_w_ | 15.67 | **<0.05** | 2.43 | 11.68 | **<0.05** | 1.68 | 6.20 | **<0.05** | 7.92 | 7.44 | **<0.05** | 3.63 |
| Isolate × ϴ | 13.73 | **<0.05** | 2.13 | 14.90 | **<0.05** | 2.14 | 1.21 | 0.27 | 1.55 | 6.35 | **<0.05** | 3.10 |
| Residual |  |  | 0.16 |  |  | 0.14 |  |  | 1.28 |  |  | 0.49 |

**Supplementary Table 6 : ANOVA results of the *F. tricinctum* growth parameters (𝜏, K, r and Vmax).** The value of Fisher’s coefficient (F), the p-value (Pr(>F)) and the proportion of variance explained (% Var) by the effect of isolate, environmental factors (a_w_ and ϴ) and their interactions (a_w_ × ϴ, Isolate × a_w_ and Isolate × ϴ) are reported for each growth parameters of *F. tricinctum*. Pr(>F) highlighted in bold indicates the significance of the Fisher test.

|  | τ | | | K | | | r | | | Vmax | | |
| --- | --- | --- | --- | --- | --- | --- | --- | --- | --- | --- | --- | --- |
| Factor | F | Pr(>F) | % Var | F | Pr(>F) | % Var | F | Pr(>F) | % Var | F | Pr(>F) | % Var |
| Isolate | 127.09 | **<0.05** | 5.53 | 22.52 | **<0.05** | 9.40 | 11.75 | **<0.05** | 14.29 | 8.81 | **<0.05** | 12.96 |
| a_w_ | 166.67 | **<0.05** | 7.25 | 122.10 | **<0.05** | 50.95 | 27.56 | **<0.05** | 33.52 | 10.03 | **<0.05** | 14.76 |
| ϴ | 1,921.17 | **<0.05** | 83.59 | 42.26 | **<0.05** | 17.63 | 15.83 | **<0.05** | 19.25 | 31.53 | **<0.05** | 46.40 |
| a_w_ × ϴ | 46.41 | **<0.05** | 2.02 | 40.29 | **<0.05** | 16.81 | 5.09 | **<0.05** | 6.19 | 4.07 | **<0.05** | 5.99 |
| Isolate × a_w_ | 8.21 | **<0.05** | 0.36 | 5.02 | **<0.05** | 2.09 | 19.96 | **<0.05** | 24.27 | 9.48 | **<0.05** | 13.95 |
| Isolate × ϴ | 27.89 | **<0.05** | 1.21 | 6.46 | **<0.05** | 2.70 | 1.04 | 0.41 | 1.27 | 3.04 | **<0.05** | 4.47 |
| Residual |  |  | 0.04 |  |  | 0.42 |  |  | 1.22 |  |  | 1.47 |

**Supplementary Table 7 : Bayesian Information Criterion (BIC)** **of the different censored ANOVA models for mycotoxin production of the five *Fusarium* species.** BIC value presented the effect of environmental factors (a_w_ and ϴ) and their interaction (a_w_ × ϴ) on mycotoxins produced by *F. avenaceum*, *F. graminearum*, *F. langsethiae*, *F. poae* and *F. tricinctum*. Models with significant BIC value are highlighted in bold.

| Species | Factor | BIC |
| --- | --- | --- |
| *F. graminearum* (TCTB) | ϴ | 932.5 |
|  | a_w_ | 757.5 |
|  | **Both factors** | **749.6** |
|  | Both + interaction | 758.4 |
| *F. langsethiae* (TCTA) | ϴ | 770.9 |
|  | a_w_ | 438.7 |
|  | **Both factors** | **429.9** |
|  | Both + interaction | 454.5 |
| *F. poae* (TCTA) | ϴ | 859.2 |
|  | **a_w_** | **775.4** |
|  | Both factors | 834.8 |
|  | Both + interaction | 791.8 |
| *F. avenaceum* (ENN) | ϴ | 704.2 |
|  | **a_w_** | **699.0** |
|  | Both factors | 736.9 |
|  | Both + interaction | 714.6 |
| *F. tricinctum* (ENN) | ϴ | 1,049.3 |
|  | a_w_ | 1,063.1 |
|  | Both factors | 1,041.2 |
|  | **Both + interaction** | **1,014.3** |

**Supplementary Table 8 : Isolate metadata.** Five isolates of five different *Fusarium* species were analysed. Their identification name in the INRAE collection (Isolate), the supplier, their location (Origin), the plant host and the year of isolation are reported.

| **Species** | **Isolate** | **Supplier** | **Origin** | **Host** | **Year** |
| --- | --- | --- | --- | --- | --- |
| *F. graminearum* | I156 | MycSA | France - 18 | Bread wheat | 2001 |
|  | I159 | MycSA | France - 18 | Bread wheat | 2001 |
|  | I164 | MycSA | France - 76 | Bread wheat | 2002 |
|  | I178 | MycSA | France - 27 | Bread wheat | 2002 |
|  | I181 | MycSA | France - 27 | Bread wheat | 2002 |
| *F. poae* | I72 | 2253 | France - 26 | Bread wheat | NA |
|  | I474 | NA | France - 28 | Wheat | 2007 |
|  | I491 | NA | France - 86 | Wheat | 2007 |
|  | I488 | NA | NA | Wheat | 2007 |
|  | I484 | NA | France - 59 | Wheat | 2007 |
| *F. langsethiae* | I509 | NA | France - 28 | Wheat | 2007 |
|  | I508 | NA | France - 28 | Wheat | 2007 |
|  | I500 | NA | France - 02 | Wheat | 2007 |
|  | I502 | NA | France - 59 | Wheat | 2007 |
|  | I466 | IFBM  E4880715 | NA | NA | NA |
| *F. avenaceum* | I873 | Canadian Collection of Fungal Cultures (FaLH27) | Canada | Wheat | 2011 |
|  | I498 | Bayer | NA | Wheat | 2007 |
|  | I874 | Canadian Collection of Fungal Cultures (FaLH03) | Canada | Wheat | 2011 |
|  | I612 | GEVES  01.01.09.06 | Scotland | Wheat | 2010 |
|  | I495 | Bayer | France - 86 | Wheat | 2007 |
| *F. tricinctum* | I104 | 2298 | France - 45 | Maize | 2001 |
|  | I106 | 2488 | France - 45 | Maize | 2001 |
|  | I524 | NA | NA | Wheat | 2007 |
|  | I526 | NA | NA | Wheat | 2007 |
|  | I86  MUCL  18522 | NA | Europe, Belgium, Wallonie, Brabant Wallon, Nil-Saint-Vincent | Barley | 1972 |
